# Evaluation of the Potential of Zero Charge of Thin‐Film (111) Platinum at the Nanoscale Using Scanning Electrochemical Cell Microscopy

**DOI:** 10.1002/smsc.70378

**Published:** 2026-08-22

**Authors:** Alejandro E. Perez‐Mendoza, Moonjoo Kim, Rico Zehl, Emmanuel Batsa Tetteh, Ellen Suhr, Alfred Ludwig, Wolfgang Schuhmann, Corina Andronescu

**Affiliations:** ^1^ Chemical Technology III Faculty of Chemistry University of Duisburg‐Essen Duisburg Germany; ^2^ Analytical Chemistry – Center for Electrochemical Sciences (CES) Faculty of Chemistry and Biochemistry Ruhr University Bochum Bochum Germany; ^3^ Chair for Materials Discovery and Interfaces Institute for Materials Faculty of Mechanical Engineering Ruhr University Bochum Bochum Germany; ^4^ CENIDE Center for Nanointegration University of Duisburg‐Essen Duisburg Germany

**Keywords:** double layer, hydrogen evolution reaction, potential of zero charge, Pt, scanning electrochemical cell microscopy

## Abstract

The potential of zero charge (PZC) is a key descriptor of the electrochemical interface, influencing ion distributions, mass transport, and reaction thermodynamics. It depends on the local surface structure, including grain orientation and atomic steps, making spatially resolved measurements essential. Scanning electrochemical cell microscopy (SECCM) enables local probing of electrochemical properties, including PZC. However, PZC probing via SECCM has been limited to pipette diameters above 1 µm due to low transient charging currents near the PZC, often at the instrumental detection limit. Here, PZC measurements on a Pt (111) thin film are demonstrated using submicron pipettes (~500 and ~250 nm diameters). Under conditions where the interfacial response is dominated by double‐layer charging, the measured PZC is consistent with macroscale results, with values of ~0.30 V versus standard hydrogen electrode (SHE). The study reveals the impact of preconditioning, electrolyte pH, and gas atmosphere used during SECCM measurements on the recorded PZC at the nanoscale on Pt (111). In particular, variations in preconditioning potential lead to systematic shifts in both PZC and hydrogen evolution reaction (HER) activity, reflecting the correlation between both properties. Overall, this work establishes a robust experimental protocol for PZC mapping at the submicron scale using SECCM.

## Graphical abstract

The local potential of zero charge of a polycrystalline Pt thin film with (111) texture was measured at the submicron level using scanning electrochemical cell using an optimized protocol, demonstrating precise assessment of the potential of zero (free) charge under well‐controlled experimental conditions.

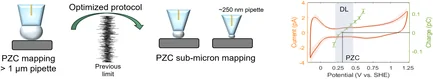

## 1 Introduction

The electrode–electrolyte interface structure plays an important role in charge transfer processes. Electrostatic interactions at the electrode–electrolyte interface, arising from surface charging, the presence of adsorbed species, and the electrochemical double layer (EDL) composition, significantly influence both the reactant behavior and the structure of interfacial water, thus having a direct impact on the electrocatalytic activity. For instance, the structure and dynamics of interfacial water strongly affect the kinetics of the hydrogen evolution reaction (HER) [1, 2, 3]. Therefore, probing the properties of the electrochemical interface may offer complementary descriptors to the adsorption energy for understanding HER activity.

The potential of zero charge (PZC) is a fundamental property of the electrochemical interface, defined as the potential at which the electrode surface, in contact with a certain electrolyte, has zero excess electron charge [4]. At potentials higher than the PZC, the surface is charged positively, while at lower potentials, it is negatively charged, leading to a reorganization of the EDL and a modification of the interfacial electric field. Therefore, the difference between the PZC and the HER equilibrium potential provides a measure of the interfacial electric field strength under operating conditions, which in turn influences the interfacial water structure and the transport of protons to the surface, directly affecting the HER kinetics. Accordingly, the PZC value has been previously used to rationalize activity trends in HER, supporting that the relative position of the PZC to the HER equilibrium potential is correlated with both interfacial water structure [1] and driving force for proton transport [5].

However, PZC measurement is nontrivial as specific ion adsorption and surface heterogeneity complicate an unambiguous identification of the PZC. This issue is particularly evident for Pt, one of the most extensively studied electrocatalyst, for which reported values of PZC under similar conditions vary considerably. For instance, scanning electrochemical cell microscopy (SECCM) studies report values of 0.65 V versus standard hydrogen electrode (SHE) for polycrystalline Pt films in 10 mM HClO_4_ [5] and 0.70 V versus SHE for polycrystalline Pt in 1 mM HClO_4_ [6]. In the macroscale experiments, several values were reported for Pt (111) surfaces using different methods: the immersion method yielded a PZC of 1.1 V versus SHE for Pt in 100 mM HClO_4_ [7], the CO displacement method provided values around 0.3 V versus SHE for Pt in HClO_4_ electrolytes with pH ranging from 1.2 to 5 [8, 9], while measurements based on differential capacitance indicate that the PZC values are 0.37 V versus SHE for Pt in 1 mM HClO_4_ [10, 11] and ~0.30 V versus SHE for Pt in 0.1 mM HClO_4_ [12, 13]. This wide variability reflects the complexity of Pt electrolyte interfaces, where hydrogen and hydroxide adsorption, ion‐surface interactions and water adsorption can all contribute to the measured charge response thereby obscuring the purely electrostatic component of the surface charge and complicating an unambiguous identification of the PZC [8, 13].

For that reason, two definitions are proposed depending on the processes taking place between the electrode and the electrolyte [4, 14, 15]: the potential of zero free charge (PZFC) where only the free electronic net charge at the electrode surface is zero, and the potential of zero total charge (PZTC) where the sum of the free electronic net charge and the charge from adsorbed species is zero. Specifically adsorbed species are ions or molecules that interact with the electrode surface through chemisorption or partial charge transfer, thereby contributing to the interfacial charge even in the absence of a well‐defined Faradaic current induced by an electrochemical reaction. When Pt is probed in an acidic electrolyte (e.g., HClO_4_) containing a nonspecifically adsorbing anion (e.g., ClO_4_^−^), the potential window of ideal polarization is restricted to approximately 0.4–0.6 V versus RHE [16], corresponding to ~0.22–0.42 V versus SHE at pH ≈ 3. Within this range, the Pt surface exhibits negligible specific adsorption, and only under these conditions, the electrochemically determined PZC value becomes meaningfully related to the intrinsic zero‐charge condition of the metal electrode surface [8, 9].

Additionally, it was suggested that the Pt PZC value is modulated by local structural features [6, 17, 18, 19, 20]. For example, variations in the grain orientation [6] and the presence of atomic steps [17, 18, 19, 20] lead to measurable shifts in PZC. Interestingly, stepped surfaces exhibit lower PZC values than Pt(111) surfaces [18, 19], which was attributed to their lower local surface potential. These findings highlight the importance of assessing the localized PZC to better understand its relationship with surface structure and electrocatalytic activity. However, the PZC has been determined for the entire electrode surface using approaches such as the capacitance method [10, 11, 12], the CO charge displacement method [8, 9], optical methods [21], and the laser‐induced temperature‐jump method [22]. Only a few studies have employed local approaches such as chronoamperometry with SECCM [5, 6] and local electrochemical impedance spectroscopy [23].

SECCM enables local electrochemical probing of a surface with different spatial resolutions, ranging from µm to tens of nm [24, 25] depending on the size of the nanopipette used and the hopping distance. A nanopipette having a hanging electrolyte droplet is brought in contact with the surface to be investigated, and upon contact, an electrochemical cell is formed, enabling electrochemical measurements to be conducted localized only in the contacted region, at given time. This enables the mapping of different electrochemical properties and, following successful colocalized structural characterization, allows correlation with the local structure or crystal orientation [25, 26, 27, 28]. However, the PZC determination using SECCM has been limited to pipettes larger than 1 µm [5, 6] due to the small transient current recorded near the PZC that is used as a stop criterion during the approach, which, if not detected, can lead to the crashing of the glass nanopipette into the surface to be investigated and thus to the failure of the experiment.

Herein, we aimed to further decrease the size of the nanopipette to the submicron level (500 and 250 nm) to enable PZC determination using SECCM with increased lateral resolution. To achieve this, we propose an adapted measuring protocol to circumvent cases in which the stop criterion for the nanopipette during the approach relies on the transient currents recorded near PZC, which are often below the detectable currents according to the signal‐to‐noise ratio of the SECCM instrument. Extending SECCM‐based PZC measurement to submicron‐sized probes enables the investigation of individual microstructural features, including submicron grains with distinct phases or crystallographic orientations, and individual catalyst nanoparticles. This capability provides new opportunities to correlate local PZC values with local structure and catalytic activity at previously inaccessible length scales.

In addition, we identified key experimental parameters influencing SECCM‐based PZC measurements on Pt, including electrolyte composition, atmospheric conditions, and interfacial contributions, that were not discussed in previous studies [5, 6]. These effects may become increasingly significant at reduced probe dimensions. Therefore, this analysis provides a framework for reliable PZC determination using submicron probes and for consistent interpretation of nanoscale electrochemical measurements on Pt surfaces.

## 2 Results

Motivated by the discrepancies in the PZC values reported on Pt surfaces using SECCM, we re‐evaluated the PZC of Pt, using a Pt (111) thin film as a model surface, that was synthesized by radio frequency (RF) magnetron sputtering followed by annealing at 900 °C for 24 h. The Pt thin film that was deposited on a Si/SiO_2_ was evaluated in terms of surface morphology and microstructure using X‐ray diffraction (XRD), scanning electron microscopy (SEM), atomic force microscopy (AFM), and electron backscatter diffraction (EBSD). Characterization of the sample via XRD, prior to and following heat treatment, revealed a pure Pt thin film with face‐centered cubic (fcc) lattice. The peaks from the 1D diffractograms were matched to #1324328 (cF4, Fm‐3m) of Pearson’s Crystal Data (PCD) (Figure S1). Although the microstructure in the as‐deposited state shows a preferred (111) orientation, indicated by high (111) peak intensity relative to the (200), (220), and (311) reflections in the 1D diffractogram, a broader orientation distribution was evidenced by multiple Debye–Scherrer ring segments in the 2D image (Figure S1a). After annealing, the (200), (220), and (311) ring segments were no longer detected, leaving only a discrete (111) arc in the merged 2D frame. Furthermore, the 1D pattern shows an approximate fivefold increase in the (111) peak intensity, indicating a strongly enhanced (111) texture in the annealed state (Figure S1a).

SEM images show significant grain growth resulting from the annealing, which is consistent with the intensity increase of the (111) peak. The grain size transitioned from a distribution below 100 nm in the as‐deposited state to a range spanning several hundred nanometers to several micrometers after annealing (Figure S1b,c). EBSD analysis confirms a preferential (111) orientation of the grains parallel to the surface normal (*z*‐direction), while the orientations in the *x*‐ and *y*‐directions remain random. Color‐coded inverse pole figure (IPF) maps are given in the Figure S2. These results, together with the XRD results after annealing, provide evidence of a strong (111) texture. Surface characterization via AFM yielded a root‐mean‐square (RMS) roughness (*S*_q_) of 0.63 nm and an average roughness (*S*_a_) of 0.44 nm. A 2D height map, 3D surface rendering, and representative surface profile are depicted in Figure S3.

**FIGURE 1:**
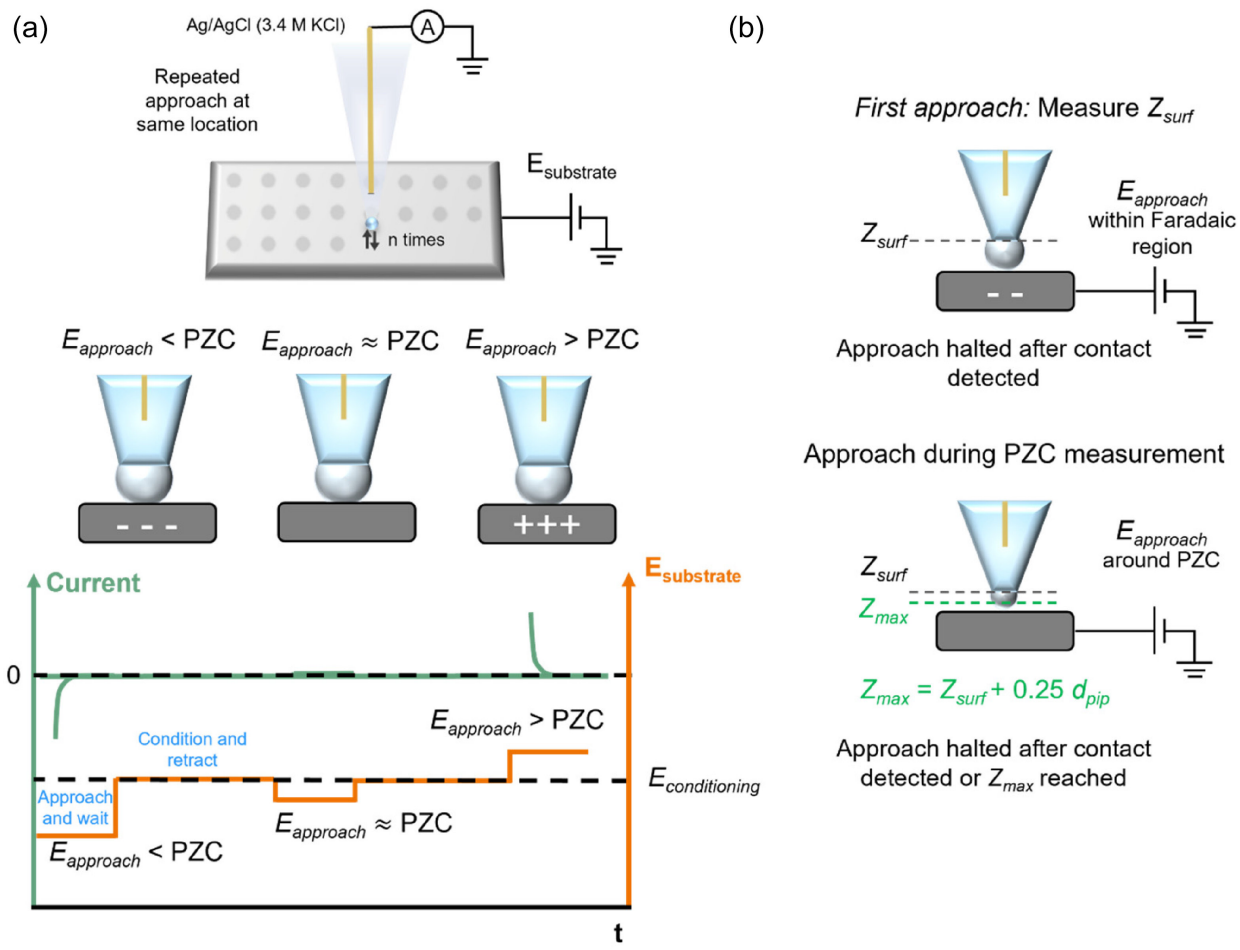
(a) Scheme illustrating the scanning electrochemical cell microscopy (SECCM) setup and the principle of PZC measurements using several repeated approaches at a number (*n*) of different potentials. (b) Scheme illustrating the criteria for maintaining the approach distance during PZC measurements to preserve the pipette integrity even when the current used as a stop criterion is lower than the signal‐to‐noise ratio of the SECCM instrument.

The PZC measurements on the (111) Pt thin film, using SECCM were performed following a similar procedure to previously described ones [5, 6], in which modifications to the approach of the nanopipette to the surface were implemented. As illustrated in Figure 1a, the electrolyte‐filled glass nanopipette, having a hanging electrolyte droplet at its tip is approached toward an electrode surface polarized at a defined potential. Upon contact between the droplet and the surface, a transient current arises due to capacitive or Faradaic currents occurring at the approach potential (*E*_approach_), which is used as stop criteria during the approach. Under these conditions, the polarity of the recorded current depends on the difference between *E*_approach_ and PZC: a positive current is observed at the approach potentials higher than the PZC, while a negative current develops at potentials lower than PZC, while at a potential equal to PZC, the recorded current approaches zero (see Figure 1a). To determine the PZC, the approach step is repeated multiple times (*n*) at different *E*_approach_, over a potential range with values more positive and more negative than the PZC. The resulting currents are integrated to obtain the transferred charge, which is further used to determine the potential at which the net charge is zero, by fitting the charge‐potential data and extracting the potential at which the fitted line crosses zero charge.

The magnitude and time constant of the transient current depend on both the size of the working electrode (i.e., the droplet footprint) and the difference between *E*_approach_ and the PZC. The challenge arises when approaching at potentials close to PZC, where no Faradaic processes occur, and thus the sole current that develops is a capacitive one, which, if generated only over a nm‐sized area, is not high enough to reach the stop criterion, leading to the failure of the experiment. Therefore, previous PZC determination conducted using SECCM used pipettes with a tip diameter below 1 µm, which enables higher currents to be recorded at the *E*_approach_ close to the PZC. In our protocol, we decoupled the stop criterion from the magnitude of the current and instead used a defined value for the surface height (*Z*_surf_) that is used during the consecutive approaches at different potentials. To achieve this, an additional step was added prior to the PZC measurement (Figure 1b, First approach) that enables the determination of the surface height (*Z*_surf_) by performing a first approach at a potential that drives a Faradaic reaction (e.g., in the HER potential window). During the subsequent PZC measurement, the motion of the pipette along the *Z* axis is limited to a maximum travel distance (*Z*_max_) that does not exceed the previously determined *Z*_surf_ by more than one quarter of the pipette diameter (Figure 1c). This threshold ensures reliable contact detection even in the presence of droplet‐size fluctuations.

Although the methodology was developed and validated using a Pt thin film with low surface roughness (*S*_q_ = 0.63 nm), the methodological advance does not rely on an atomically smooth surface, but on decoupling surface localization from PZC determination through an initial determination of the surface height. As in conventional single‐barrel SECCM, performance on rougher surfaces will ultimately depend on the ability to accurately position the nanopipette above the local surface.

Following this, the steps already used in previous protocol [5] are used, including the potentiostatic conditioning step applied to remove adsorbed species (e.g., H^+^ or OH^−^) followed by the pipette retraction and a sequence of new approaches conducted at different potentials around the expected PZC. Initially, the conditioning potential (*E*_conditioning_) was set to 0.29 V versus SHE, which lies in the double‐layer region of Pt and may restore the double‐layer structure while ensuring a reproducibly clean surface state. Additional details of the PZC measurement, data processing, and examples of approach curves are provided in the Section 4 and Figures S4–S6. In addition, a linear sweep voltammetry was performed after PZC measurement from −0.150 to −0.510 V versus SHE at 0.1 V s^−1^ to evaluate the HER activity and drive correlations it with the PZC values.

### 2.1 PZC of Pt at Different HClO_4_ Concentrations

There is a general agreement that the PZFC of Pt lies between 0.23 and 0.30 V versus SHE, as indicated by the CO displacement method, which accounts for specific adsorption contributions, as well as by indirect methods such as laser temperature‐jump [19] and optical methods [21]. These values indicate that the PZFC can be most reliably assessed electrochemically in electrolytes with pH values close to 3, where it is located near the center of the potential window in which the interfacial response of Pt is predominantly capacitive. At pH values further from this condition, deviations arise as the interfacial response of Pt departs from ideal capacitive behavior due to the concurrent contributions from specifically adsorbing species in solution [8, 9]. Since the SECCM‐based method does not allow the separation of the contributions of free and specific adsorption charges, we report all measured values simply as PZC, following the convention in previous electrochemical studies. In addition, although the electrode exhibits a strong (111) texture (as confirmed by structural characterization), it remains a polycrystalline thin film with finite grain size and surface heterogeneity, which are expected to broaden or attenuate ideal Pt(111)‐type electrochemical signatures [29, 30]. In this context, faradaic processes such as oxygen reduction, if trace oxygen is present, as well as incipient adsorption of H/OH species, may overlap with the capacitive current, thereby influencing the apparent zero‐charge condition.

**FIGURE 2:**
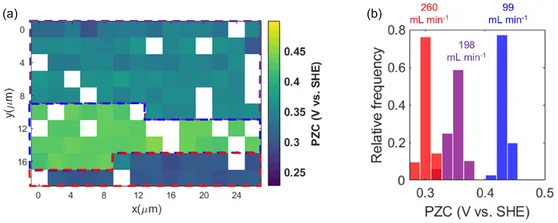
PZC recorded on the Pt (111) in 10 mM HClO_4_ under varying Ar flow (99 (blue), 198 (purple), 260 mL min^−1^ (red)). (a) PZC map showing the regions where different Ar flow was used, enclosed by colored lines. (b) Histogram showing the distribution of the PZC grouped by Ar flow. The number of measurements considered for experiments conducted at Ar flow rates of 99, 198, and 260 mL min^−1^ were 22, 53, and 17, respectively.

To assess the influence of oxygen reduction on the measured PZC, the atmosphere was controlled by placing the sample inside a chamber supplied with humidified Ar through a mass flow controller, a configuration previously demonstrated to provide effective control of the local atmosphere [31]. The experiments were conducted using a pipette bigger than 1 µm filled with 1 mM HClO_4_ (see Figure S7 and Table S1) and different Ar‐flow, and we observed that the change in Ar‐flow has a strong impact on the calculated PZC, with a positive shift of the PZC being observed as the Ar flow was decreased (Figure 2), suggesting that additional reactions take place, one of them being the O_2_ reduction reaction.

The presence of oxygen when a flow of 99 mL min^−1^ was used is also evident from the capacitive region in the Pt voltammogram (see Figure S8a). An increase of the gas flow to 198 and 260 mL min^−1^ leads to a flattening of the currents recorded in the capacitive region between 0.3–0.4 V versus RHE, without a significant difference being observed between the two high flow rates. The presence of O_2_ when a lower Ar flow is used can be also noticed from the charge derived from the transient current recorded after approaching at different *E*_approach_. We observed that a higher negative charge is obtained at lower Ar flow compared to the higher ones, as depicted in Figure S8b. The results indicate that enough Ar‐flow needs to be ensured in our environmental chamber to ensure that the recorded currents are not a result of unwanted reactions in the capacitive region, one of them being the oxygen reduction reaction, if O_2_ is still present in the surrounding atmosphere of the formed electrochemical cell. Based on these results, all subsequent experiments were carried out using a flow of 260 mL min^−1^ Ar to minimize the impact of O_2_ on the measurements.

**FIGURE 3:**
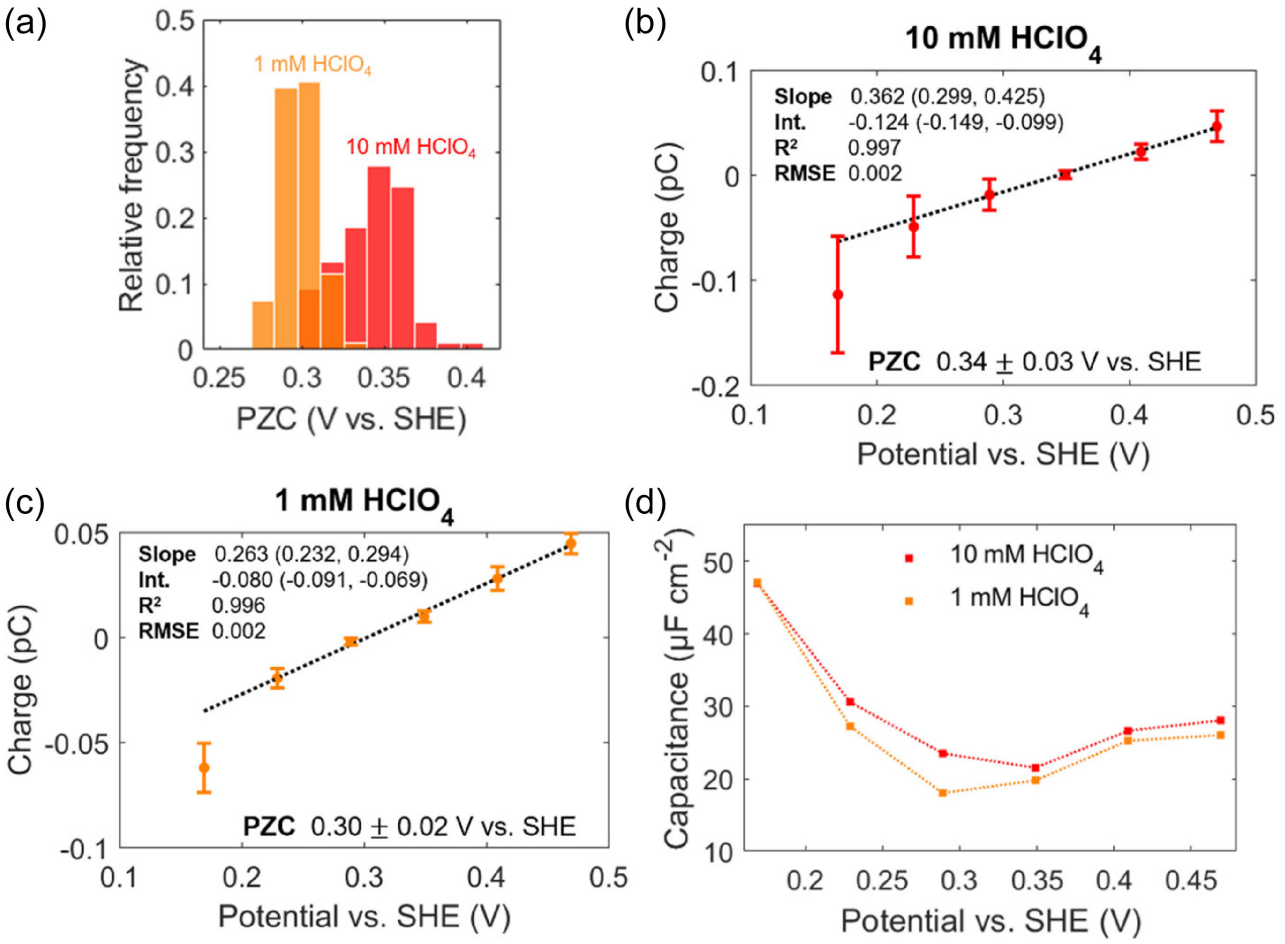
(a) Histogram showing the distribution of PZC on a Pt (111) evaluated in 10  (red) and 1 mM (orange) HClO_4_ electrolytes, the averaged charge calculated as a function of the electrode potential for the measurements performed in (b) 1 mM and (c) 10 mM HClO_4_ and the capacitance calculated with the estimated charges and the SECCM tip opening area (d). The numbers of measurements considered were 97 and 96, respectively. Error bars indicate standard deviations. Dotted lines show linear regressions excluding potential points within the H adsorption region on Pt.

As previously discussed, the PZC of Pt can also be influenced by specific adsorption occurring during the measurements. Therefore, we conducted experiments using a pipette with a diameter higher than 1 µm filled with 10 or 1 mM HClO_4_ electrolyte (pH 2 and pH 3). Under those experimental conditions used and based on literature reports, we expected the impact of the possible adsorption of hydrogen and hydroxide on the measured potentials to be minimized, since the potential range around the PZC lies predominantly within the nominal double‐layer regime of Pt [8, 12]. The potential window (0.17–0.47 V vs. SHE) was selected to encompass the expected PZC of Pt, covering potentials lower and higher than the nominal PZC value while maintaining the measurement largely within the capacitive region of the Pt response under the chosen electrolyte conditions [8, 12]. As shown in Figure 3a, the calculated PZC values are around 0.3 V versus SHE, with a mean value of 0.299 ± 0.002 V versus SHE for 1 mM and 0.341 ± 0.004 V versus SHE for 10 mM HClO_4_. The difference between the two values is attributed to an increased contribution of hydrogen adsorption processes within the potential window used for the analysis in the 10 mM electrolyte, which partially overlaps with the hydrogen adsorption region of Pt and modifies the apparent charge‐potential response used for PZC determination.

The presence of H^+^ adsorption is evidenced by the deviations from linearity in the charges calculated at 0.169 and 0.229 V versus SHE for 10 mM HClO_4_ (Figure 3b) and at 0.169 V versus SHE for 1 mM HClO_4_ (Figure 3c). The aforementioned potentials lie within the hydrogen adsorption region of Pt for the respective electrolytes, and hence the corresponding charges likely include a contribution from H^+^ adsorption. Additionally, the specific capacitance estimated from the charging current during each approach, and normalized by the tip opening area, ranges from 20 to 30 µF cm^−2^ within the double‐layer region, with a minimum close to 0.30 V versus SHE as observed in Figure 3d, consistent with previously reported values for Pt [11, 32]. In contrast, the capacitance at 0.169 V versus SHE is approximately twice as high as that at other potentials, further supporting that the measured current at lower potentials likely includes a contribution from hydrogen adsorption, as expected to occur at this potential on Pt [12, 13].

As discussed above, hydrogen adsorption is dictated by the approach potential. Although the potentiostatic pretreatment step is designed to remove residual ions adsorbed before establishing a new electrolyte–surface contact at the same location, some adsorbed species may remain on the Pt surface, inducing a shift in the apparent PZC. Therefore, in the next step we verified the effect of different conditioning potentials employed during the potentiostatic pretreatment step on the determined PZC in 1 mM HClO_4_, by using potentials in the double‐layer region (0.32 V vs. SHE), the H‐adsorption region (0.10 V vs. SHE), and the region where OH adsorption is expected (0.50 V vs. SHE). As shown in Figure S9a, when conditioning was applied in the H‐adsorption region, the resulting PZC decreased to 0.19 V versus SHE, whereas conditioning in the OH adsorption region increased the PZC to 0.48 V versus SHE. The average charge versus potential plot shows a noticeable deviation from linearity at potentials above 0.50 V versus SHE (Figure S9b–d), which may be associated with the formation of OH and O layers on the Pt surface. Interestingly, when a conditioning potential of 0.520 V versus SHE was applied, the charges derived at 0.260 and 0.340 V versus SHE were significantly more negative than expected from the linear trend, with the reductive transient becoming stronger when a larger potential step relative to the Pt redox potential is applied. Thus, we could show that the determined PZC values follow literature trends, where theoretical studies have shown that adsorption of OH and O species induces inward‐oriented interfacial dipoles, which stabilize electrons at the surface [33, 34], increasing the work function and thereby shifting the PZC toward more positive values [33]. Conversely, one can expect that the presence of partial hydrogen coverage withdraws electronic charge from the Pt surface, rendering it relatively more positive [33, 35] and the interplay between these effects may change the apparent PZC of the Pt surface. The above‐mentioned effects could also account for the higher measured PZC values in 10 mM HClO_4_. Previous studies have demonstrated that OH adsorption from water dissociation can occur on Pt steps even at relatively low potentials, between 0.07 and 0.27 V versus SHE in a pH  ~ 3 electrolyte [36], supporting that even at this low concentration, OH may be absorbed on the surface.

Altogether, the PZC measurements yield a value in line with most reported values for PZC of Pt (~0.3 V vs. SHE [8, 9, 12, 22]), particularly when 1 mM HClO_4_ electrolyte (pH 3) was used. These experiments highlight the importance of considering how experimental conditions and the applied potential range influence transient surface structures when interpreting the calculated PZC values. Because the potential region in which the interfacial charge of Pt is purely capacitive is limited, the PZC can be directly assessed using the SECCM methodology presented here only under specific electrolyte conditions, typically corresponding to pH values around 3, where the PZC lies near the center of the double‐layer potential region. Moreover, potentiostatic conditioning in the double‐layer region at around 0.32 V versus SHE minimizes surface modifications associated with the presence of residual adsorbed species. The presence of surface charge may shift the apparent PZC; therefore, only when the Pt surface is sufficiently free of adsorbates can the measured value be considered representative of the PZFC. In addition, the choice of potential range for measurements is critical to avoid overlap between capacitive and pseudocapacitive processes, as electrochemical methods inherently have both contributions to the measured charge.

### 2.2 Measurement of PZC With Smaller Pipettes

**FIGURE 4:**
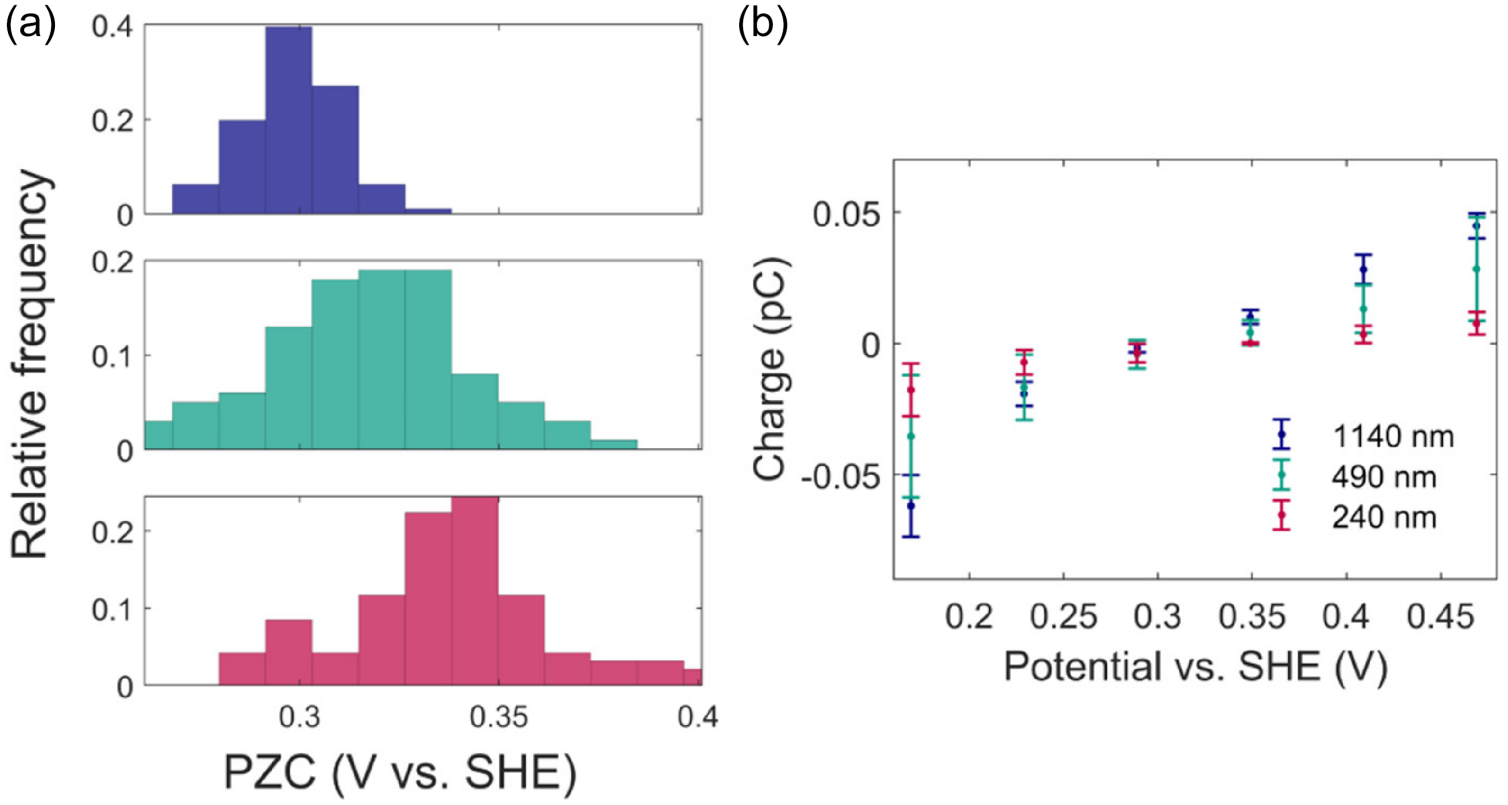
(a) Histogram showing the distribution of the potential of zero total charge (PZC) of a Pt (111)‐textured surface in contact with 1 mM HClO_4_ electrolyte using different SECCM tip sizes 1140 (blue), 490 (green), and 240 nm (red) and the (b) averaged charge as a function of the electrode potential. The numbers of measurements used for the graphs were 96, 100, and 94, respectively. Standard deviations are represented by error bars.

After establishing the measurement conditions for Pt with the ~1 µm pipettes, we measured the PZC using smaller ones (490 and 240 nm) also in 1 mM HClO_4_ (see heatmaps with local PZC in Figure S10). We observed that as the pipette size decreased, PZC values became more widely distributed and the mean shifted to higher values, as shown in the histogram in Figure 4a. As expected, the charges recorded at each potential using smaller nanopipettes also decreased. Interestingly, while the slope of the charge versus potential plot decreased with the size of nanopipettes, the recorded PZC values remain between 0.3–0.35 V versus SHE, consistent with the PZC value recorded with the µm‐sized nanopipettes. Still, when decreasing the size of the nanopipette, we observe an increasing variation of the PZC values. The median PZC increased from 0.299 V for the 1140 nm nanopipette to 0.337 V for the 240 nm nanopipette, while the median absolute deviation increased from 0.0078 to 0.0134 V and the standard deviation increased from 0.0113 to 0.0254 V (see Table S2). This increased variability may be partly attributed to an increased sensitivity to local surface heterogeneity. As the pipette size decreases, the measurement becomes progressively less spatially averaged, leading to a stronger contribution from defects and crystallite domain edges. In contrast, larger pipettes average over a wider surface area, thereby smoothing out local variations, as previously suggested [37]. This interpretation is supported by AFM measurements (Figure S11) performed on regions in the proximity of the SECCM scanned areas, which reveal grain‐scale surface features with lateral dimensions comparable to the nanopipette dimensions. This indicates that reducing the probe size increases sensitivity to such local morphological variations. Additionally, the lower signal obtained around the PZC can also contribute to the observed spreading due to a reduced signal‐to‐noise ratio, which becomes more pronounced for smaller pipettes.

For the 240 nm pipette, transient currents after the approach at 0.289 and 0.349 V versus SHE were frequently not recorded or had values lower than 2 pA, being very close to the noise level of our instrument (Figure S12a). At potentials of 0.169 and 0.469 V versus SHE, which are further from the PZC, the transient currents reached a maximum of only ~10 pA followed by a rapid decay. Conversely, in the experiments using the 1140 or 490 nm pipettes, even at potentials close to the PZC (0.289 and 0.349 V vs. SHE), a current of a few pA could still be measured (Figure S12b and c). Consequently, PZC calculation with the 240 nm pipette relied primarily on currents recorded farther from the actual PZC, which may lead to higher errors in the PZC estimation, since the calculation relies only on detectable transient currents that, in this case, happen only at significantly different potentials than the PZC. Nevertheless, the change in current polarity could still be clearly identified, allowing a reasonable estimation of the PZC. Based on these observations, we expect that PZC determination with pipettes even smaller than 240 nm is limited using the current protocol and instrumentation.

Further analysis shows that the charges, and hence the capacitance, estimated with the 240 nm pipette are larger than expected for Pt double‐layer charging (Figure S13a) for which capacitances between 20 and 100 µF cm^−2^ are reported. From the slope of the plot of average charge versus applied potential (Figure S13b–d) for the different SECCM nanopipette diameters, the apparent specific capacitances are 131 ± 20 , 85 ± 7, and 25 ± 2 µF cm^−2^ for the 240, 490, and 1140 nm tips, respectively. The higher apparent specific capacitance values measured with the smaller pipette are likely to arise from other experimental factors rather than intrinsic changes in the Pt double‐layer. First, the capacitances are normalized using the pipette aperture area; therefore, some uncertainty in the reported values should be expected, as the true electrochemically contacted area is determined by the meniscus footprint. However, estimations of the wetted area based on hydrogen adsorption charge (Figure S14 and Table S3) suggest that the contacted area remains of the same order of magnitude as the pipette aperture area and does not totally account for the observed changes on the estimated specific capacitance. Additionally, contributions from the instrumental capacitance associated with the pipette walls or measurement circuitry are expected [38] and may become nonnegligible when the currents are too small. The highly confined meniscus geometry may further promote partial overlap of double layers at the meniscus perimeter, increasing the apparent capacitance [39, 40]. Together, these effects can explain the systematically larger capacitances obtained with decreasing pipette size.

We estimated the instrumental capacitance from the transient current recorded after the potential was changed to start a new approach at the same location. When no contact between the droplet and the surface is established (open circuit), the current recorded upon the potential change may be associated only with the measurement circuitry and the pipette [38]. The instrumental capacitance derived for a 240 nm pipette was around 0.025 ± 0.001 pF (Figure S15a). Whereas, the total capacitance measured when the pipette was in contact with the surface was 0.069 ± 0.005 pF (Figure S15b). So, it can be expected that a significant overestimation of capacitance occurs when smaller pipettes are used. Dividing the estimated instrumental capacitance by the tip area, it would be as large as ~54 µF cm^−2^ for the 240 nm pipette.

Nevertheless, even though the absolute charge may be overestimated, the change in current polarity could still be clearly identified for the 240 nm pipette, allowing the determination of the PZC. The precision of the PZC determination with this approach is primarily limited by the minimum potential step relative to the actual PZC required to drive a detectable charging current, rather than by the absolute accuracy of the measured charge. For the 240 nm pipette, this potential step exceeds 0.08 V. Based on these observations, one can expect an acceptable PZC estimation of Pt‐based surfaces using pipettes as small as 240 nm, and we anticipate that accurate PZC determination using pipettes that are significantly smaller will be increasingly challenging under the present experimental protocol and instrumentation. This study, therefore, served to partially define the practical limits of PZC measurement with SECCM, showing that reliable determination is feasible down to ~250 nm, while highlighting the challenges of pushing to smaller scales.

### 2.3 Examining HER Currents and the Relationship With the PZC

**FIGURE 5:**
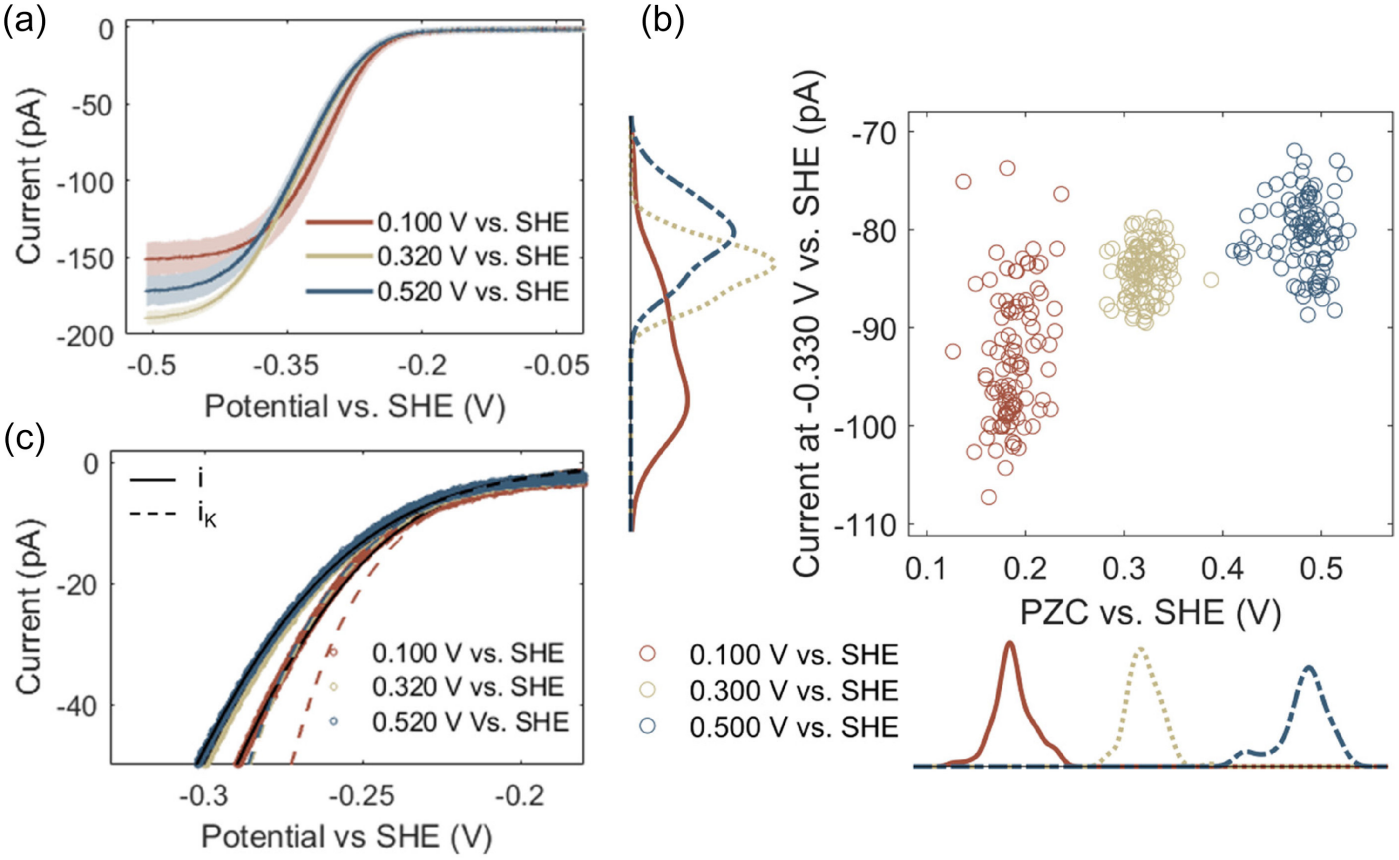
(a) LSVs recorded to evaluate the PZC and local HER activity on a (111) Pt thin film in 1 mM HClO_4_. Data was acquired at 0.1 V s^−1^ after potentiostatic conditioning at 0.100 (dark red) 0.320 (yellow) and 0.520 V (dark blue) versus SHE. Shaded regions represent ±1.96 standard deviations. (b) Scatter plot of HER current measured at −0.330 V versus SHE as a function of PZC, including data from experiments performed under different conditioning potential. (c) Zoomed‐in view of voltammograms in (a) showing the measured current (colored symbols), the kinetic current (dashed lines) and the total current predicted by model (solid black line).

After establishing the effects of different experimental conditions on the PZC and discussing the practical limits of PZC determination using SECCM, we next examine how the PZC correlates with the HER activity of the Pt surface, which was evaluated using linear sweep voltammetry from −0.150 to −0.51 V versus SHE at 0.1 V s^−1^. Varying the potentiostatic pretreatment potential produces significant shifts in both the PZC values and HER currents. In contrast, within a single experiment conducted at a fixed conditioning potential, the PZC and HER values remain essentially unchanged. This is illustrated in Figure 5b, which presents the current recorded at −0.330 V versus SHE plotted against the estimated PZC values.

The dependence on conditioning potential immediately prior to HER evaluation is particularly noteworthy. As discussed above, when the applied conditioning potential is lowered into the hydrogen adsorption region of Pt, the measured PZC of the Pt in 1 mM HClO_4_ system shifts to lower values. Simultaneously, an increase in HER current is observed at lower overpotentials, in the range −0.200 to −0.380 V versus SHE, as shown in Figure 5a. Overall, a correlation between the PZC and the HER current is observed across different conditioning potentials, as illustrated by the scatter plot of the HER current measured at −0.330 V versus SHE versus the corresponding PZC (Figure 5b). This relationship suggests that the conditioning potential induces changes in the surface state of Pt that affect both its electrostatic properties and catalytic activity. Importantly, these surface modifications are not fully reversed during the subsequent potential sweep used to evaluate the HER, indicating a memory effect of the conditioning step on the surface chemistry.

To rationalize the observed trends, we consider the surface states induced by different conditioning potentials. Conditioning at 0.100 V versus SHE lies within the hydrogen adsorption region and is expected to induce a significant coverage of adsorbed hydrogen. Conditioning at 0.320 V versus SHE corresponds closely to the PZC of the Pt/HClO_4_ interface, so the surface is expected to be largely free of specifically adsorbed species. In contrast, conditioning at 0.520 V versus SHE lies in the potential region where hydroxyl adsorption and the initial formation of oxygen‐containing species on Pt become favorable. Although during the subsequent cathodic sweep the surface will again reach significant hydrogen coverage, the observed differences suggest that the modifications produced during potentiostatic conditioning are not fully reversed. In this context, preadsorbed hydrogen enhances the HER rate, likely by increasing the local reactant concentration, whereas the presence of oxygenated species suppresses HER, consistent with partial site blocking and modified interfacial energetics. This effect may be particularly pronounced at the low electrolyte concentration used (1 mM HClO_4_), where the diffusion process plays a dominant role in the electrochemical response. Under these conditions, changes in the surface state induced by the conditioning potential may have an amplified impact on the measured currents.

To further assess the kinetic contribution ($i_{k}$) we fitted the HER response using a model proposed in the literature [41] (see Equation (5) in the Section 4). This approach allows the extraction of the transfer coefficient *α* and the so‐called exchange current $i_{0}=n\cdot F\cdot A\cdot k_{0}\cdot C_{b}$, from which the kinetic current was calculated as $i_{k}=\text{−}n\cdot F\cdot A\cdot k_{0}\cdot C_{b}\cdot \exp(-\alpha \cdot f\cdot (E-E_{0}))$. It should be noted that the absolute magnitude of $i_{k}$ depends on the effective wetted area, which cannot be determined independently in the present experiments and may vary with the conditioning potential. Consequently, the higher $i_{k}$  values observed following the conditioning within the hydrogen adsorption region may partially reflect changes in the wetted area in addition to changes in the intrinsic HER kinetics. However, the trend observed in α, a value which is independent of electrode area, provides additional evidence for a kinetic effect. In particular, surfaces conditioned at 0.100 V versus SHE exhibit the highest values, indicating stronger dependence of the reaction rate on the applied potential, and therefore, more favorable HER kinetics. Furthermore, this conditioning potential does not yield the highest limiting current, suggesting that the observed enhancement in HER activity cannot be attributed solely to an increase in the contacted area.

The transfer coefficient *α* shows a value close to 1 for the surfaces conditioned at 0.100 V versus SHE, compared with α≈0.85 for more positive conditioning potentials. A higher α indicates a stronger sensitivity of the reaction rate to the applied potential, which can arise from a surface state that facilitates proton reduction. For HER on Pt in acidic media, it is considered that if the Volmer step (proton electrosorption) is the rate‐determining step (RDS), the apparent transfer coefficient is close to 0.5, whereas if the Heyrovsky step (electrochemical desorption) is considered as RDS, the apparent transfer coefficient should be 1.5 when the H adsorbed is in quasi‐equilibrium and the coverage of active sites is close to unity [42, 43]. Thus, the intermediate values observed here suggest a mixed kinetic regime. This behavior is consistent with deviations from quasiequilibrium and with variations in surface hydrogen coverage. Taken together, these observations indicate that the shifts in the PZC are correlated with systematic changes in HER activity, predominantly reflecting modifications of the Pt surface and adsorption state rather than a purely electrostatic effect.

## 3 Conclusions

The SECCM‐based PZC measurements presented here provide insights into the experimental conditions required to ensure that the measured PZC reflects solely the capacitive charging, and is clearly distinguished from the PZTC, which includes both capacitive and pseudocapacitive contributions. Under these conditions, the obtained values are consistent with literature‐reported PZC of Pt (~0.3 V vs. SHE) in acidic electrolyte (1 mM HClO_4_, pH ≈ 3), confirming the reliability of the approach when the interfacial response is governed primarily by double‐layer charging. Moreover, the results reveal that variations in preconditioning potential lead to systematic changes in both PZC and HER activity. When the preconditioning potential promotes hydrogen adsorption, HER activity at low electrolyte concentration (1 mM HClO_4_) is enhanced, highlighting the correlation between PZC and HER activity, which in this case is governed by surface adsorption. Additionally, the analysis of transient currents across different pipette sizes demonstrates that, while the PZC can still be determined from the current polarity change even with sub ‒300 nm probes, the quantitative accuracy decreases as the pipette diameter is reduced. This limitation stems from the challenges associated with measuring the current at the nanoscale and with the instrumentation, which impedes the precise capture of fast charging transients. Consequently, reliable PZC determination using pipettes smaller than ~240 nm will likely require enhanced measurement instrumentation and/or modified experimental protocols optimized for nanoscale electrochemical transients.

## 4 Experimental Procedures

### 4.1 Preparation and Structural Characterization of Pt Thin Film

Using a five‐cathode magnetron sputtering system (DCA Finland, cathodes confocally mounted and aimed at the substrate center), equipped with a 100 mm diameter Pt target (FHR, 99.990% purity) a 150 nm thick Pt thin film was synthesized using Ar (99.9999 % purity) to sputter Pt at 0.67 Pa onto the substrate by using a power density of 2.22 W/cm^2^ (RF) under constant 10 rpm rotation of an oxidized 4‐inch diameter Si wafer with a 500 nm SiO_2_‐layer. This oxide layer acts as a diffusion barrier, preventing the formation of Pt silicide upon annealing, cf [44]. Before the deposition, the chamber’s base pressure was at 5 · 10^−6^ Pa and the Pt target was sputter‐cleaned with Ar ions for 100 s at 0.74 W/cm^2^ and 2.67 Pa.

After synthesis, the initial sample was partitioned into smaller specimens. A designated specimen was annealed in a tube furnace (Thermconcept, ROK 150/500/12) under vacuum in the range of 10^−5^ Pa at 900 °C for 24 h to obtain larger grains and a well‐defined (111) texture. Still under vacuum, the sample cooled down until extraction and further characterization.

XRD analyses, before and after the annealing of the Pt thin film, were performed with a Bruker D8 Discover equipped with a Vantec‐500 2D detector in Bragg–Brentano geometry with an Incoatec High Brilliance Iµs Cu Kα X‐ray source (*λ* = 0.15418 nm). Three frames were taken before and after annealing, respectively, at *θ*/2*θ* pairs of 12.5°/25°, 22.5°/45°, and 32.5°/65° with a shutter speed of 60 s for each frame. The merged 2D frames were then integrated into 1D diffractograms for peak indexing.

SEM was done with scanning electron microscope JEOL JSM‐7200F using 5 kV acceleration voltage and 5 mm working distance (WD) with a lower electron detector (LED) to optically verify grain growth and sample quality prior to AFM and EBSD.

AFM and EBSD were used to characterize the surface topography and crystallographic orientation of the Pt thin films. AFM measurements were carried out using a Bruker scanning probe microscope (Bruker) operated in ScanAsyst in Air mode, which is a PeakForce tapping mode, employing a probe with a nominal spring constant of approximately 0.4 N m^−1^. EBSD analysis was performed in a scanning electron microscope (JEOL JSM‐7200F) equipped with an EBSD detector (Oxford AZtecSymmetry CMOS detector, Oxford Instruments), and the acquired diffraction patterns were analyzed using standard orientation‐mapping software to determine the grain orientation and texture of the Pt film.

### 4.2 Protocol for Mapping the PZC

All the SECCM measurements were performed using a home‐built SECCM setup further described in the Section S4 along with the preparation of the pipettes and electrolytes used. The electrochemical measurement protocol using SECCM for this work is based on the protocol followed by previous SECCM studies [5, 6] and it is summarized in the flow diagram in Figure S4a. It began with a first approach to sense the surface height. When using 1 mM HClO_4_ electrolyte, this approach was performed on the surface polarized at −0.43 V versus Ag/AgCl (3.4 M KCl). The approach was halted using a conservative threshold current of approximately ± 6 times the standard deviation of the background noise (*σ* = 0.270 pA), which significantly exceeds the standard statistical requirement for signal detection. After the first approach, four cyclic voltammograms were recorded between 1.02 and −0.43 V versus Ag/AgCl (3.4 M KCl) at 2 V s^−1^, which served to clean the surface prior to the PZC measurement protocol. An example of a voltammogram is shown in Figure S4b.

The PZC measurement protocol began with a potentiostatic conditioning step, during which the surface was held at 0.08 V versus Ag/AgCl (3.4 M KCl) for 500 ms to remove adsorbed hydrogen and oxygen species before the next approach of the tip. Then the SECCM tip was retracted and approached again to the surface polarized at a predetermined potential *E*_approach_. After meniscus contact, the transient charging current was recorded during 150 ms, sufficient to capture both the current peak and exponential decay. The conditioning and approach steps were repeated six times at the same location, varying *E*_approach_ from −0.03 to 0.27 V versus Ag/AgCl (3.4 M KCl) in equidistant increments. An example of the recorded signals is shown in Figure S4c. After the PZC measurement a cyclic voltammogram was recorded between 0.1 and −0.65 V versus Ag/AgCl (3.4 M KCl) at a scan rate of 0.1 V s^−1^. This step was performed to evaluate the local HER activity.

After this final step, the pipette was retracted and moved to a new location to build electrochemical maps covering multiple points on the surface. The mapping was performed using the so‐called scan hopping mode, in which the SECCM probe was moved from one location to another according to a predefined grid, thereby generating spatially resolved electrochemical images of the substrate. The measurements on Pt(111) thin film with different Ar flow conditions, electrolytes, conditioning potentials, and pipettes were performed over 14 × 10 individual spots.

In the absence of Faradaic processes, the moment when the droplet contacts the sample surface can be modeled as a series RC circuit. The transient current recorded during this process can be described by Equation (1), where i is the current, $V_{\mathrm{app}}$ is the applied voltage, t is the time, $R_{\mathrm{sol}}$ is the solution resistance, and $C_{\mathrm{EDL}}$ is the electrode–electrolyte interface capacitance.

(1) $$i=V_{\mathrm{app}}/R_{\mathrm{sol}}\cdot \exp\;(\text{−}t/\left(R_{\mathrm{sol}}\cdot C_{\mathrm{EDL}}\right))\;$$

The charge associated with the formation of the EDL (*Q*_EDL_) at the different approach potentials was determined by integrating the current trace up to three times the time constant (*τ*). The time constant *τ* is defined as the time required to charge a capacitor with a capacitance of $C_{\mathrm{EDL}}$, through a resistor with the resistance $R_{\mathrm{sol}}$ to approximately 63.2% of its final charge, whereas 3*τ* represents the time required to attain 99.5% of its final charge. It was calculated by fitting the initial part of the current trace to Equation (1) (see Figure S6).

To improve the numerical accuracy of the integration, the experimental current traces were interpolated to generate a denser time grid, increasing the number of data points by a factor of three prior to integration. Finally, the PZC was determined as the *x*‐intercept of a linear fit to *Q*_EDL_ versus approach potential, as shown in Figure S4d. Finally, the PZC was evaluated by the *x*‐intercept of the linear fit to *Q*_EDL_ versus approach potential as indicated in Figure S4d. For further analysis, only the measured spots with PZC values with coefficient of determination (*R* ^2^) higher than 0.95 were considered. The data from the selected spots were used to construct histograms and to estimate the mean PZC and mean charge for each condition, which is reported together with the 95% confidence interval, calculated as:

(2) $$\overset{ˉ}{x}\pm t_{0.975,\;n-1}\cdot \frac{s}{\sqrt{n}}$$

where $\overset{ˉ}{x}$ is the mean, s is the standard deviation, and n is the number of measurements, and $t_{0.975,\;n-1}$ is the Student’s *t* critical value at the 95% confidence level. n is reported in the corresponding figures showing the histograms. Additionally, a specific capacitance was calculated from the mean charge ($q_{i}$) at each *E*_approach_ ($E_{i}$), the difference between the corresponding $E_{i}$ and the mean PZC, and the area of the tip aperture ($A_{tip}$):

(3) $$C\left(E_{i}\right)=\frac{q_{i}}{\left(E_{i}-\mathrm{PZC}\right)\cdot A_{\mathrm{tip}}}$$

The PZC results are reported with respect to the SHE potential scale, as it is a property that should be independent of the electrolyte pH. Reporting on this scale facilitates discussion and comparison with values previously reported in the literature. To convert the potential recorded during SECCM measurements from the Ag/AgCl (3.4 M KCl) QRCE scale to the SHE scale, the following equations were used:

(4) $$E_{\mathrm{SHE}}[V]=E_{\mathrm{Ag}/\text{AgCl}/3.4\text{M KCl}}+0.210+E_{\mathrm{OCP}}$$

where $E_{\mathrm{Ag}/\text{AgCl}/3.4\text{M KCl}}$ is the applied potential versus the quasi‐reference counter electrode (QRCE), 0.210 V is the standard potential of the Ag/AgCl (3 M KCl) reference electrode at 25 °C, $E_{\mathrm{OCP}}$ is the measured open circuit potential difference between the QRCE and the Ag/AgCl/3 M KCl reference electrode.

The voltammograms evaluating the HER were analyzed using the analytical expression for the current (i) as function of the applied potential (E) during SECCM discussed in literature [41]:

(5) $$i(E)=\frac{n\cdot F\cdot A\cdot m\cdot C_{b}\cdot \exp\;(\text{−}\text{α}\cdot (E-E_{0}))}{\begin{array}{cc} m\text{⁄}k_{0} & +\;\exp\;(\text{−}\alpha \cdot f\cdot (E-E_{0}))\\ & +\;\exp\;((1-\alpha )\cdot f\cdot (E-E_{0})) \end{array}}$$

where α the transfer coefficient, $k_{0}$ the standard rate constant, and f=F/(R·T), where F is the Faraday constant, R the gas constant and T the temperature in Kelvin. Whereas the transport‐kinetic coupling is described by the parameter m which is defined as $m=i_{\lim}^{\text{SECCM}}/(n\cdot F\cdot A\cdot C_{b})$ where n represents the number of electrons transferred, A the effective electrode area, $C_{b}$ the bulk concentration of HClO_4_, and $i_{\lim}^{\text{SECCM}}$ the experimental limiting current.

The fitting procedure was conducted in two stages. First, a global fit was performed to determine $i_{\lim}^{\text{SECCM}}$ over the full potential range. Subsequently, a refined fit was executed in a narrower potential window (from −0.05 to −0.15 V vs. RHE) to extract the kinetic parameters α and $i_{0}=n\cdot F\cdot A\cdot k_{0}\cdot C_{b}$.

Since the experiments were conducted in 1 mM HClO_4_, an electrolyte with low ionic strength, the model accounts for both migration and ohmic drop. Migration is implicitly included in the measured $i_{\lim}^{\text{SECCM}}$, while the ohmic drop was addressed by replacing the applied potential E with the effective potential $E_{\mathrm{eff}}=E-i\cdot R_{s}$ in Equation (4). The resistance $R_{s}$ was estimated for each meniscus contact as [45]:

(6) $$R_{s}=\frac{F\cdot D_{H^{+}}\cdot C_{b}}{\kappa \cdot i_{\lim}^{\text{SECCM}}}$$

where $D_{H^{+}}$ is the H^+^ diffusion coefficient and *κ* the electrolyte conductivity. Physically, this estimation of $R_{s}$ reflects the dual nature of ion transport in binary electrolytes; the same electric field that drives the migration of H^+^, thereby enhancing the limiting current, is also responsible for the ohmic drop. By deriving from $i_{\lim}^{\text{SECCM}}$, the model ensures that the potential correction is intrinsically scaled to the specific geometry and access resistance of each meniscus contact. The implementation of this ohmic correction was critical to revealing the intrinsic kinetics of the system; while uncorrected data yielded lower apparent transfer coefficients α≈0.75, the corrected fits consistently converged to α≈1 in agreement with the Heyrovsky step as the RDS for HER on Pt. The values of parameters used for Equations (5) and (6) are provided in Table S4, whereas Table S5 summarizes the results of the data fitting to Equation (5).

## Funding

This study was supported by Deutsche Forschungsgemeinschaft (506711657 and 440951282).

## Conflicts of Interest

The authors declare no conflicts of interest.

## Supporting information

Experimental data complementing Pt thin film characterization via X‐ray diffraction (XRD), scanning electron microscopy (SEM), electron backscatter diffraction (EBSD) and atomic force microscopy (AFM) (Section S1), Supporting section about PZC measurement protocol using SECCM (Section S2), additional experimental data supporting effect of Ar flow on PZC measurement (Section S3), additional experimental data complementing PZC measurement after different conditioning potentials (Section S4), additional experimental data complementing PZC measurement with different SECCM tip sizes (Section S5), additional information about the voltammogram analysis using mathematical model (Section S6). All additional Figures and tables are referred to in the main text.

## Data Availability

The data that support the findings of this study are openly available in zenodo at https://zenodo.org, under DOI: 10.5281/zenodo.21246427. The published dataset represents an archival snapshot corresponding to the data used in this publication. The current version of the dataset, including subsequent updates and corrections, is available through the CRC 1625‐ Research Data Management System at: https://crc1625.mdi.ruhr‐uni‐bochum.de/object/acidic‐her‐activity‐potential‐of‐zero‐charge‐structurecomposition‐correlations‐in‐pt‐based‐systems‐21579.
