# Supplementary material for: Evaluation of the Potential of Zero Charge of Thin‐Film (111) Platinum at the Nanoscale Using Scanning Electrochemical Cell Microscopy
Source: Small Sci. 2026 Aug 22;6(8):e70378. doi: 10.1002/smsc.70378 (PMC13516997; doi:10.1002/smsc.70378)
Supplement: Supplementary file 1 [file SMSC-6-e70378-s001.pdf]

Supporting Information

Evaluation of the Potential of Zero Charge of Thin-Film (111) Platinum at the Nanoscale using Scanning Electrochemical Cell Microscopy

Alejandro E. Perez-Mendoza[1], Moonjoo Kim[2], Rico Zehl[3], Emmanuel Batsa Tetteh[2], Ellen Suhr[3], Alfred Ludwig[3], Wolfgang Schuhmann[2], Corina Andronescu*[1,4]

[1] Chemical Technology III, Faculty of Chemistry, University of Duisburg-Essen, Carl-Benz-Straße 199, 47057 Duisburg, Germany.

[2] Analytical Chemistry – Center for Electrochemical Sciences (CES), Faculty of Chemistry and Biochemistry, Ruhr University Bochum, Universitätsstraße 150, Bochum, Germany

[3] Chair for Materials Discovery and Interfaces, Institute for Materials, Faculty of Mechanical Engineering, Ruhr University Bochum, Universitätsstraße 150, Bochum, Germany

[4] CENIDE, Center for Nanointegration University of Duisburg-Essen; Carl-Benz-Straße 199, 47057 Duisburg, Germany

[*] Correspondence: corina.andronescu@uni-due.de (C. Andronescu)

S1. Supporting Information 1 – Pt thin films characterization

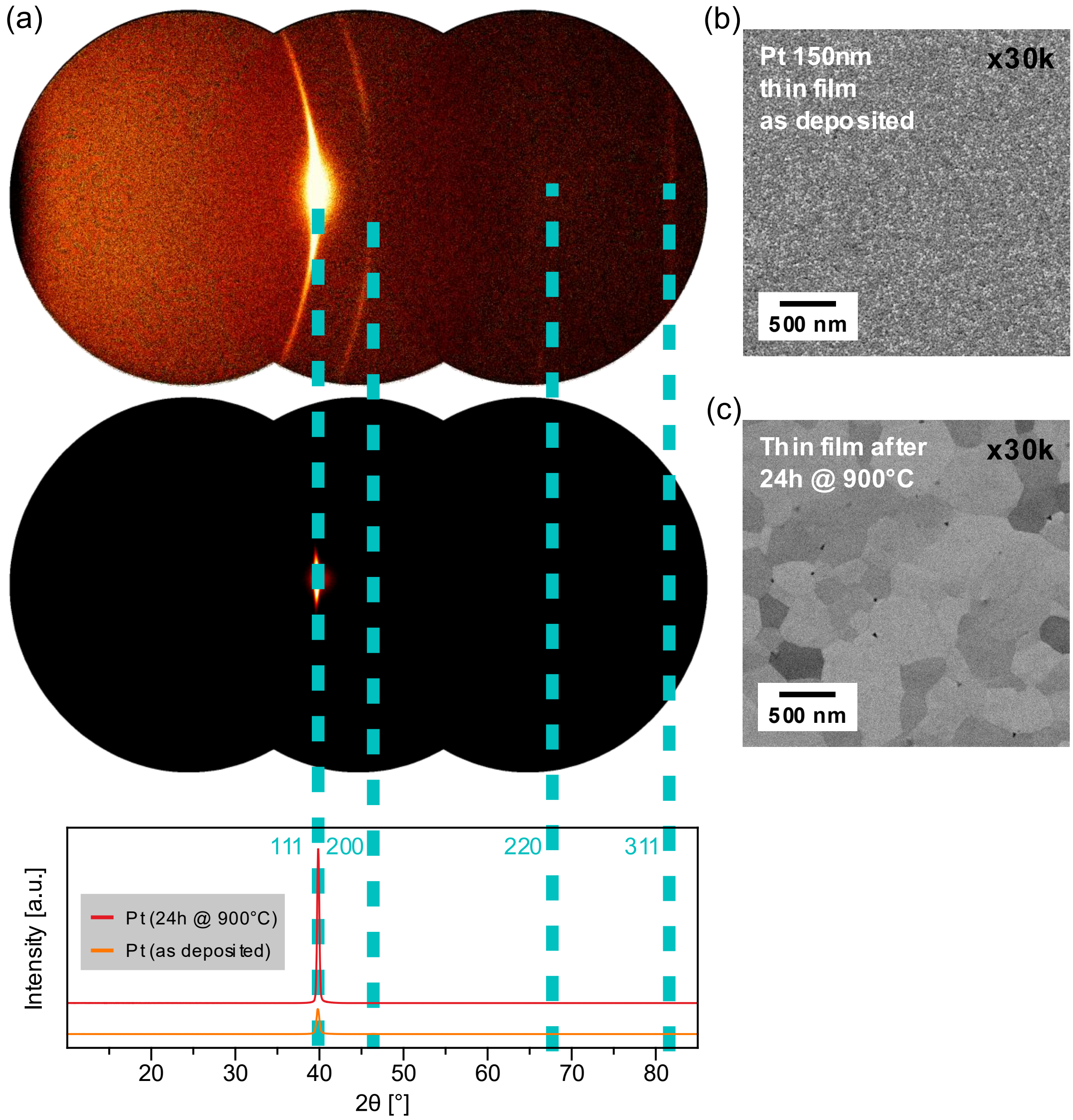

Figure S1. X-ray diffraction (XRD) patterns and scanning electron microscopy (SEM) images of the sample in the as-deposited and annealed states. (a) shows the merged 2D diffraction frames for the as-deposited (top) and annealed (middle) states, with the corresponding derived 1D diffractograms plotted at the bottom. Position indicators for the indexed peaks are superimposed on the 2D frames to facilitate comparison. (b) and (c) show SEM images of the as-deposited and annealed surfaces, respectively. Peaks were indexed according to the reference #1324328 (cF4, Fm-3m) from Pearson's Crystal Data (PCD).

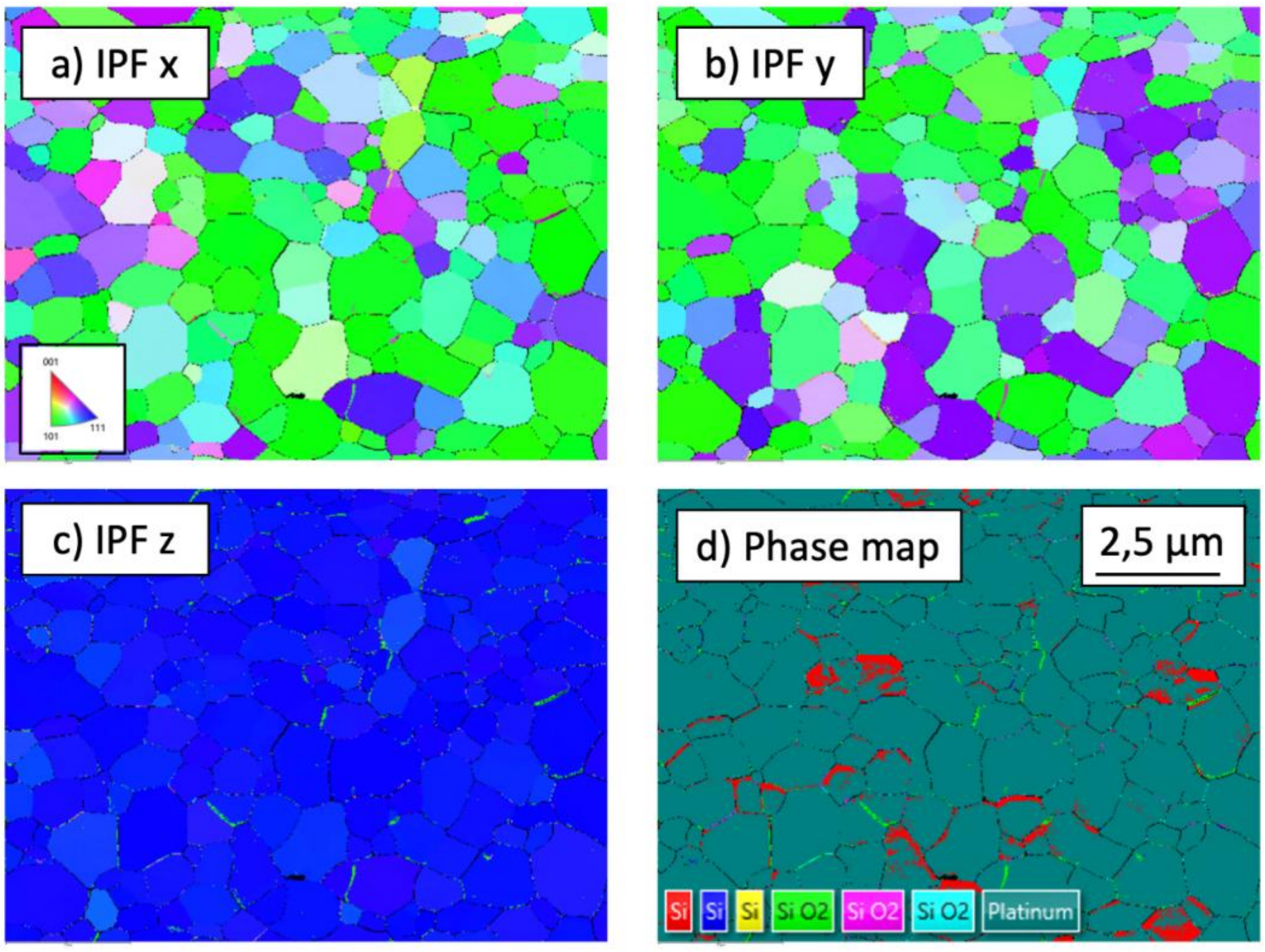

Figure S2. Inverse Pole Figure (IPF) maps of the Pt thin film: in a) x-direction, b) y-direction, c) z-direction, and d) a phase map. X- and y-directions are parallel to the substrate surface, while the z-direction is perpendicular. The phase map in d) emphasizes that there are no other phases present; the detected Si-phase represents the substrate, which was detected in the region of the grain boundaries, due to lower thin film density and thickness.

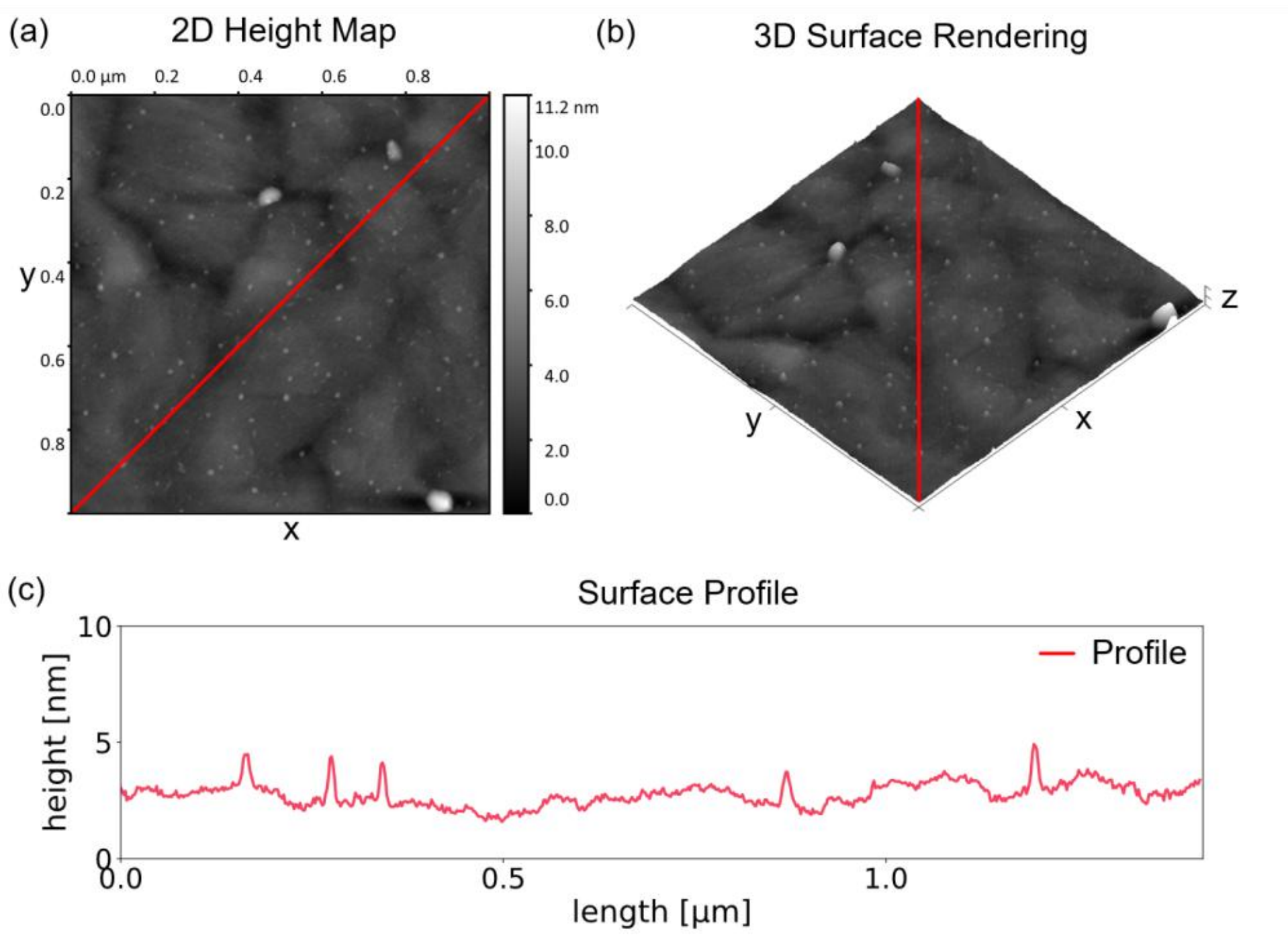

Figure S3. Results of atomic force microscopy (AFM) show a rather smooth surface with root-mean-square (RMS) roughness (S_q) 0.63 nm and an average roughness (S_a) of 0.44 nm. (a) a 2D height map 1 × 1 μm^2, (b) a 3D surface rendering of the same area and (c) the corresponding surface profile along the red line of (a) and (b).

S2. Supporting Information 2

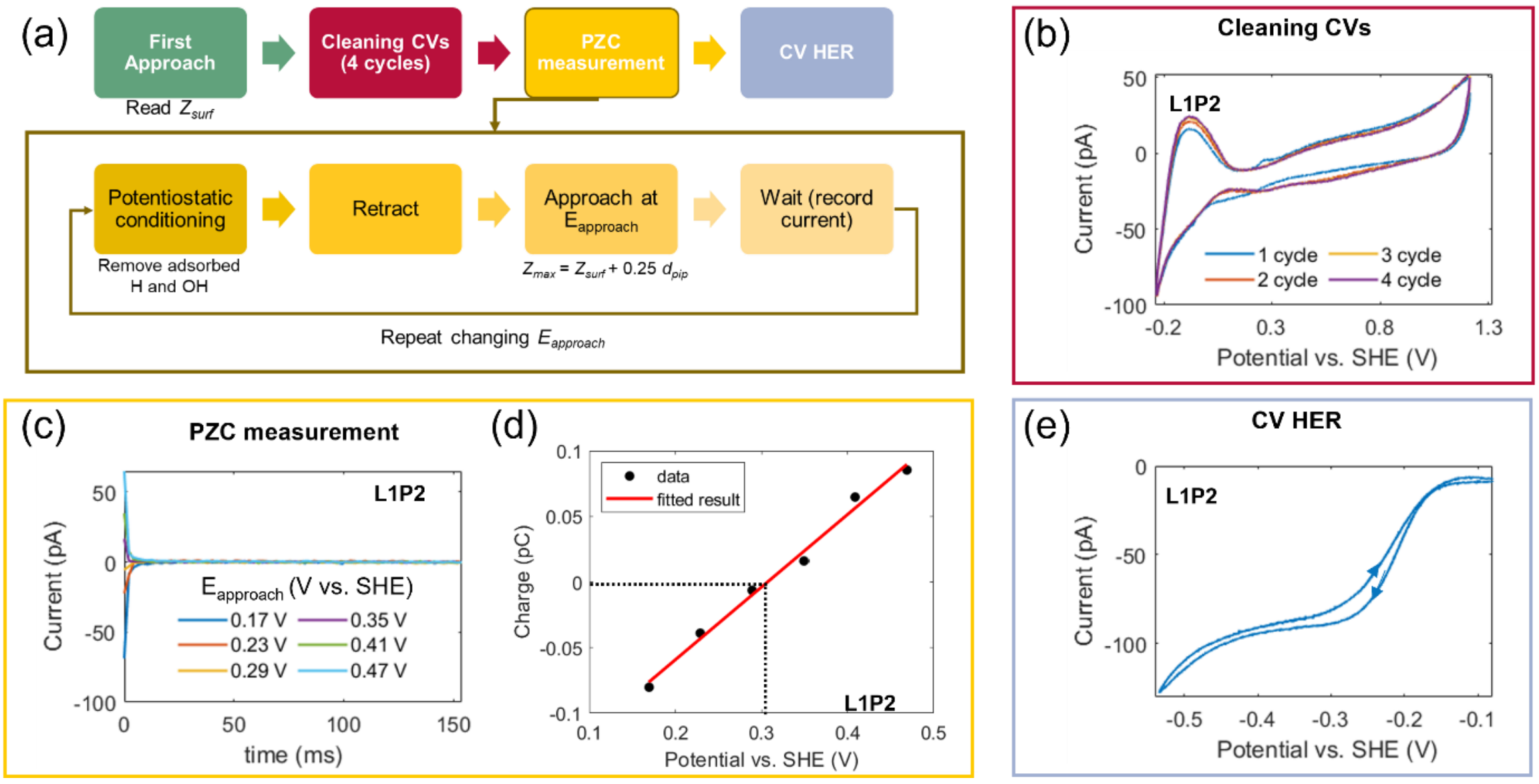

Figure S4. SECCM protocol for electrochemical measurements. (a) Schematic illustration of the measurement sequence followed at each single location, including steps to sense the surface height, clean the surface, measure the PZC through repeated approaches at different potentials, and evaluate the HER activity. (b) Example of a set of 4 cyclic voltammograms recorded at 2 V s^{-1} for surface cleaning. (c) Example of current transients recorded during PZC measurements. (d) Example of PZC determination from the charge extracted by integrating the current traces shown in (c). (e) Example of a cyclic voltammogram recorded after PZC measurements at a relatively low scan rate of 0.1 V s^{-1} for HER kinetic evaluation. The data shown correspond to the measurements performed on a Pt surface in 1 mM $HClO_4$ (discussed in Figure 3).

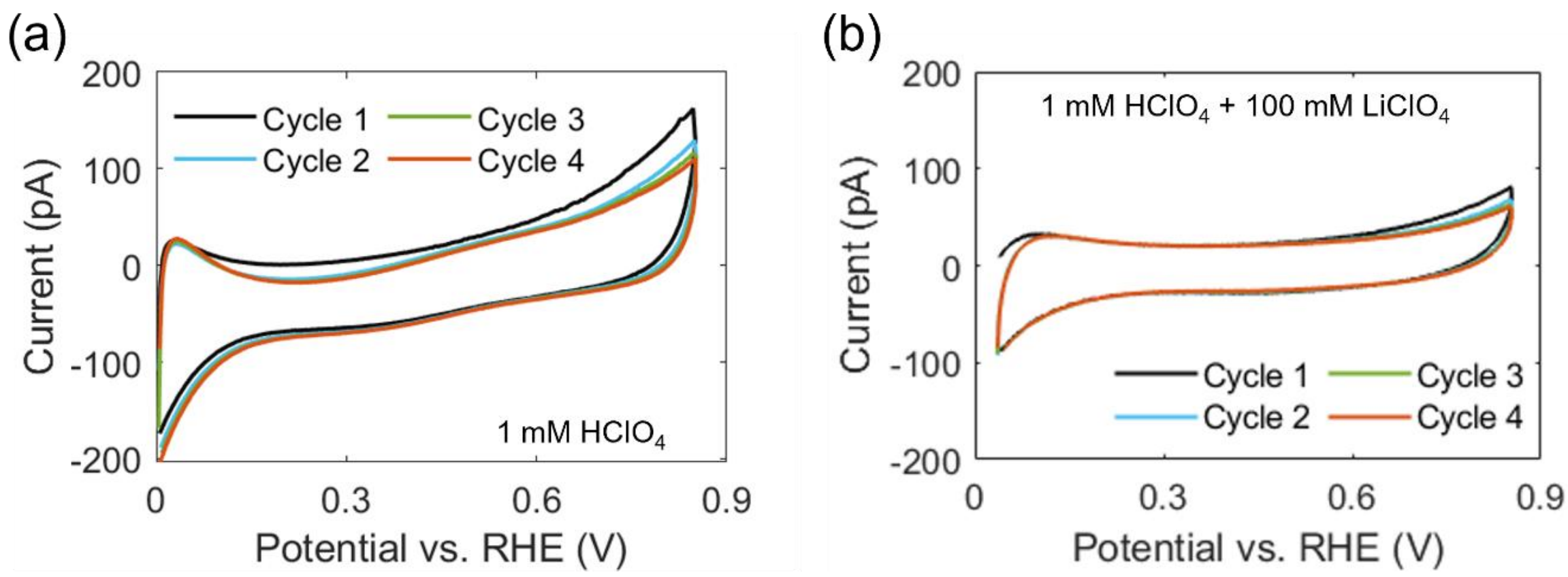

Figure S5. Voltammograms recorded between 0 and 0.85 V vs. RHE inside a glovebox (O_2 < 0.1 ppm) using a ~1 µm pipette filled with (a) 1 mM $HClO_4$ and (b) 1 mM $HClO_4$ + 100 mM $LiClO_4$. The curves correspond to the average of 30 and 18 measurements, respectively. Addition of supporting electrolyte results in a substantially flatter response in the nominal double-layer region.

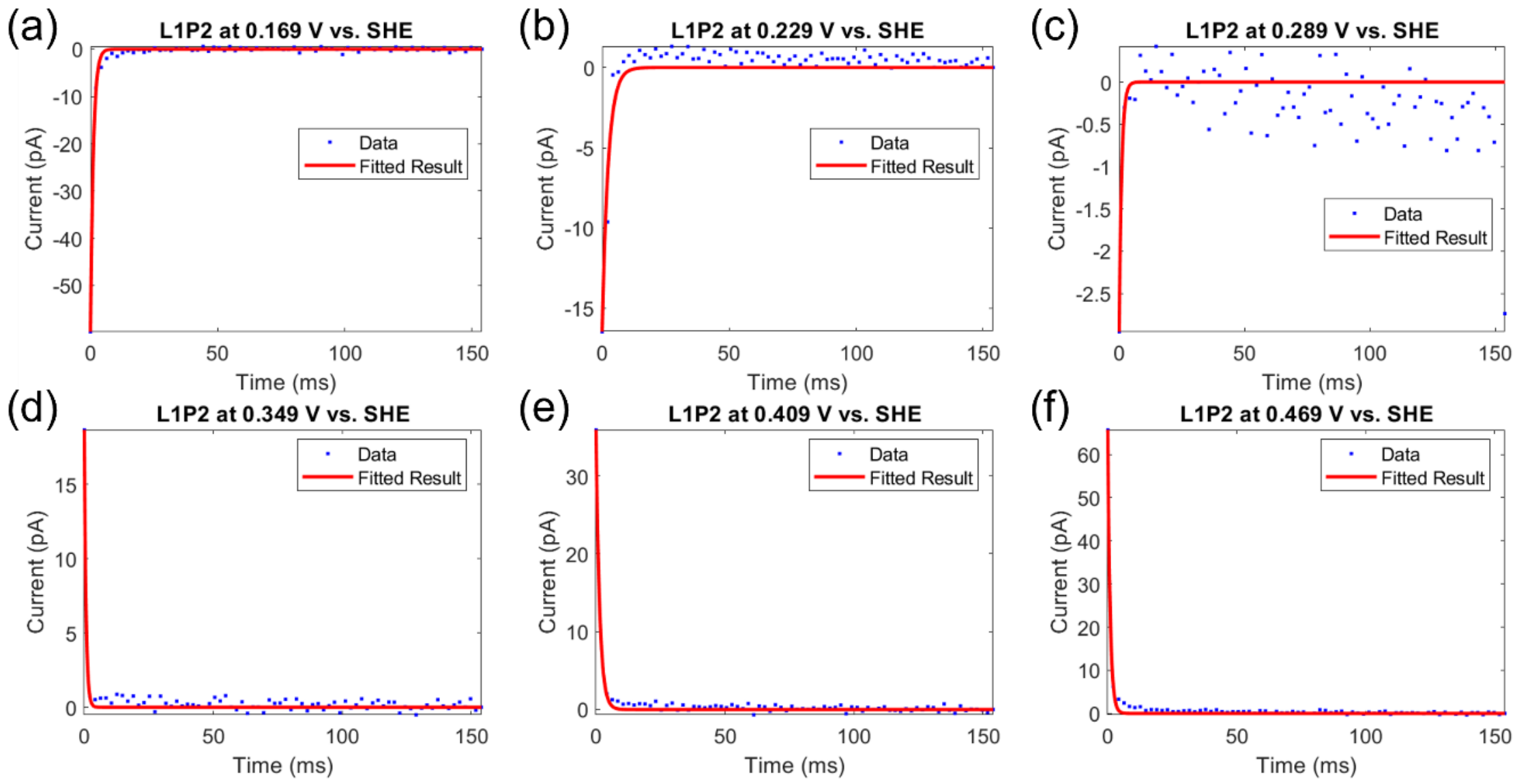

Figure S6. Example of exponential fitting of the current upon the contact at the approach potentials of (a) 0.169 V, (B) 0.229 V, (C) 0.289 V, (D) 0.349 V, and (E) 0.469 V vs. SHE. The data shown correspond to the measurements performed on a Pt surface in 1 mM $HClO_4$ (discussed in Figure 3).

Scanning electrochemical cell microscopy (SECCM) instrumental set-up

All electrochemical experiments were conducted using a home-built SECCM setup. In this configuration, a single-barrel SECCM pipette (fabrication described below) was filled with electrolyte. The pipette was mounted on a stationary holder, while the sample (polycrystalline Pt) was placed inside a chamber fixed to a sample holder attached to the positioning system. Coarse positioning of the sample was achieved using motorized precision x–y–z stages (Owis) controlled by an LStep PCIe controller (Lang).

An optical imaging system, consisting of a VZM™ 200i zoom lens (Edmund Optics) and a Motic camera, was used to assist in the initial positioning of the SECCM tip approximately 40 μm above the region of interest. Fine approach and positioning of the SECCM probe relative to the surface were performed using an x–y–z piezo positioner (P-611.3S NanoCube, Physik Instrumente) mounted beneath the sample holder. The setup was mounted on a benchtop passive vibration isolation platform (50BM-8, Minus K Technology) placed on a vibration isolation workstation (M-VIS3648-SG2-325A, Newport). The platform was enclosed by thermal insulation panels (vakuVIP B2, Vaku-Isotherm) and a bronze mesh Faraday cage (VIS-FDC-3648, Newport).

Upon contact of the electrolyte meniscus at the SECCM tip with the surface, a two-electrode electrochemical cell was formed, with the substrate acting as the working electrode and the leak-free Ag/AgCl (3.4 M KCl) electrode (0.5 mm outer diameter, Innovative Instruments) serving as both the reference and counter electrode (QRCE). The potential was

controlled with respect to ground, and the current flowing through the QRCE was measured using a variable-gain transimpedance amplifier (DLPCA-200, FEMTO Messtechnik). Data acquisition and instrument control were performed using an FPGA card (USB-7855R) and a modified LabVIEW (National Instruments) program based on the Warwick Electrochemical Scanning Probe Microscopy (WEC-SPM) toolbox. The data were acquired by averaging 1024 data points collected every 2 μs, corresponding to an effective data acquisition rate of 2048 μs.

All electrochemical experiments were conducted under an Ar atmosphere to prevent interference from O_2. For this purpose, the sample was enclosed in a chamber made of acrylonitrile–butadiene–styrene polymer wrapped in aluminium foil and continuously purged with humidified Ar at a flow rate of 260 mL min^{-1}. The gas flow was controlled using a mass flow controller (EL-Flow Select F-201CV-100, Bronkhorst Instruments), with the volumetric flow rate specified under European standard conditions (1 atm, 20 °C). The chamber was fitted with a transparent polymethyl methacrylate (PMMA) lid containing a 1 cm diameter circular aperture to allow the positioning and translational motion of the nanopipette.

SECCM Pipette Fabrication

The SECCM tip consisted in a single-barrel pipette filled with electrolyte ($HClO_4$ at different concentrations), prepared by diluting 70% $HClO_4$ (Sigma-Aldrich) with deionized water. Prior to the SECCM scan, the electrolyte was deaerated by Ar bubbling for 30 min. The single-barrel pipettes were fabricated by pulling single-barrel quartz glass capillaries (QF120-90-10, Sutter Instruments) using a CO2-laser puller (P-2000, Sutter Instruments). The used quartz capillaries were carefully cleaned with wipes soaked in ethanol. Nanopipettes were pulled using a two line program with tuned parameters to get pipettes with tip diameters of 250, 500 and 1100 nm. Those parameters are: heat (HEAT, controls the power of the laser beam), filament (FIL, controls the scanning length of the laser beam), velocity (VEL, controls the movement of both ends of the capillary before the hard pulling), delay (DEL, controls the time waited after heating and before the hard pulling), and pull (PUL, control the force of the hard pulling).

Program for pipettes of 250 nm tip diameter

Line 1	HEAT 780	FIL 4	VEL 45	DEL 130	PUL 0
Line 2	HEAT 780	FIL 4	VEL 45	DEL 130	PUL 125

Program for pipettes of 500 nm tip diameter

Line 1	HEAT 780	FIL 4	VEL 45	DEL 130	PUL 0
Line 2	HEAT 780	FIL 4	VEL 45	DEL 130	PUL 100

Program for pipettes of 1100 nm tip diameter

Line 1 HEAT 780 FIL 4 VEL 45 DEL 130 PUL 0

Line 2 HEAT 780 FIL 4 VEL 45 DEL 130 PUL 80

The "average diameter" (d) of the pipette was calculated as the geometric mean of the semi-major (a) and semi-minor (b) axes, which is equivalent to finding the radius of a circle with the same area as the ellipse ($d=\sqrt{(a\cdot b)}$). a and b were measured from the SEM images shown in Figure S7 and the results of the estimated diameters are presented in Table S1.

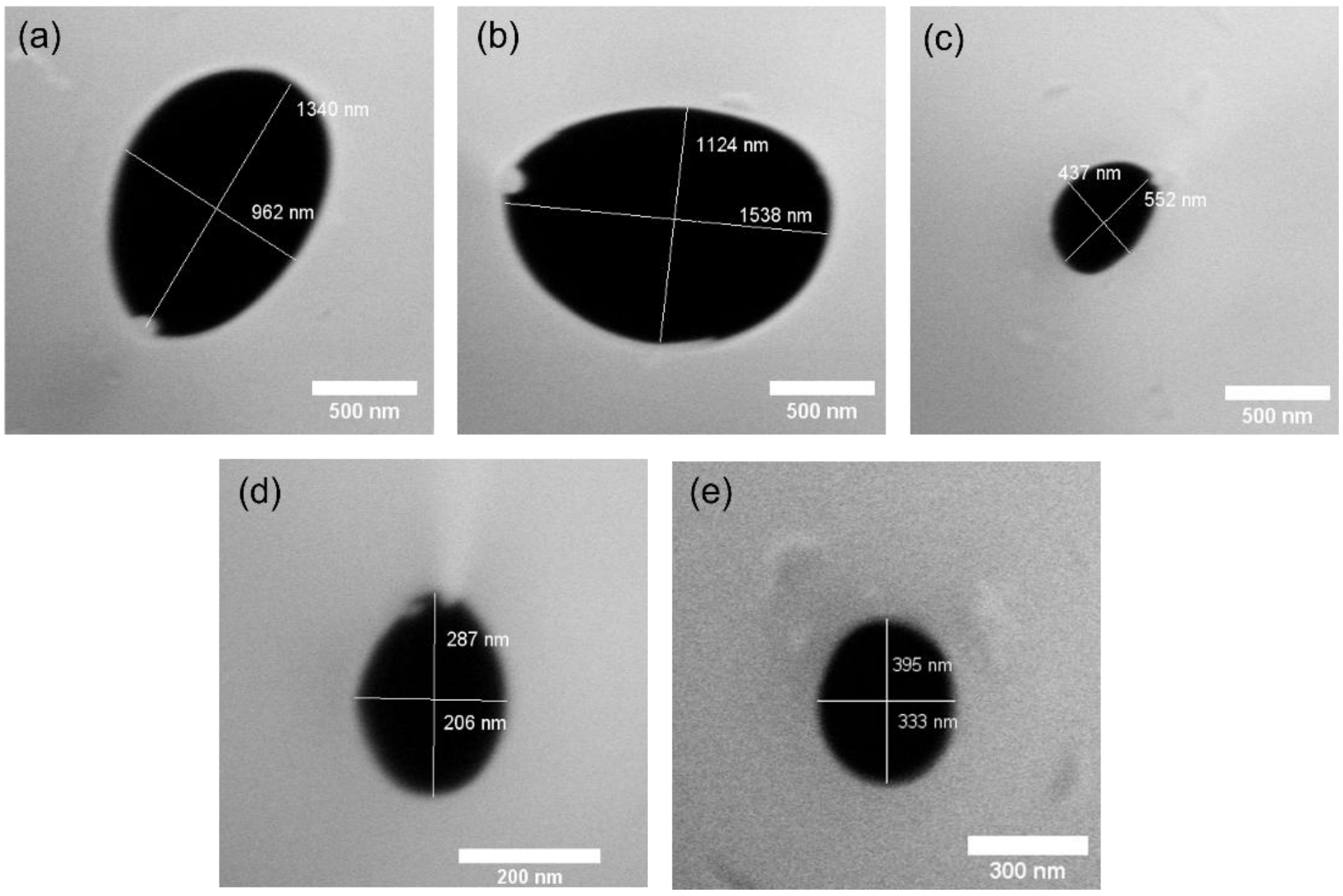

Figure S7. SEM images of the tip opening of the pulled single barrel quartz pipettes used for the PZC measurements of Pt conducted in (a) 1 mM $HClO_4$ with tip diameter higher than 1 µm (b) 10 mM $HClO_4$ with tip diameter higher than 1 µm, (c) 1 mM $HClO_4$ with tip diameter around 500 nm, (d) 1 mM $HClO_4$ with tip diameter around 250 nm and (e) 1 mM $HClO_4$ with tip diameter around 360 nm.

Table S1. Estimated average diameters of the pipettes used for the SECCM measurements.

Measurement	a (nm)	b (nm)	d (nm)
1 mM $HClO_4$, varying Ar flow	1340	962	1135
10 mM $HClO_4$	1124	1538	1315
1 mM $HClO_4$	437	552	491
1 mM $HClO_4$	287	206	243
1 mM $HClO_4$ (varying conditioning)	395	333	360

S3. Supporting Information 3 – Experiments under different Ar flow

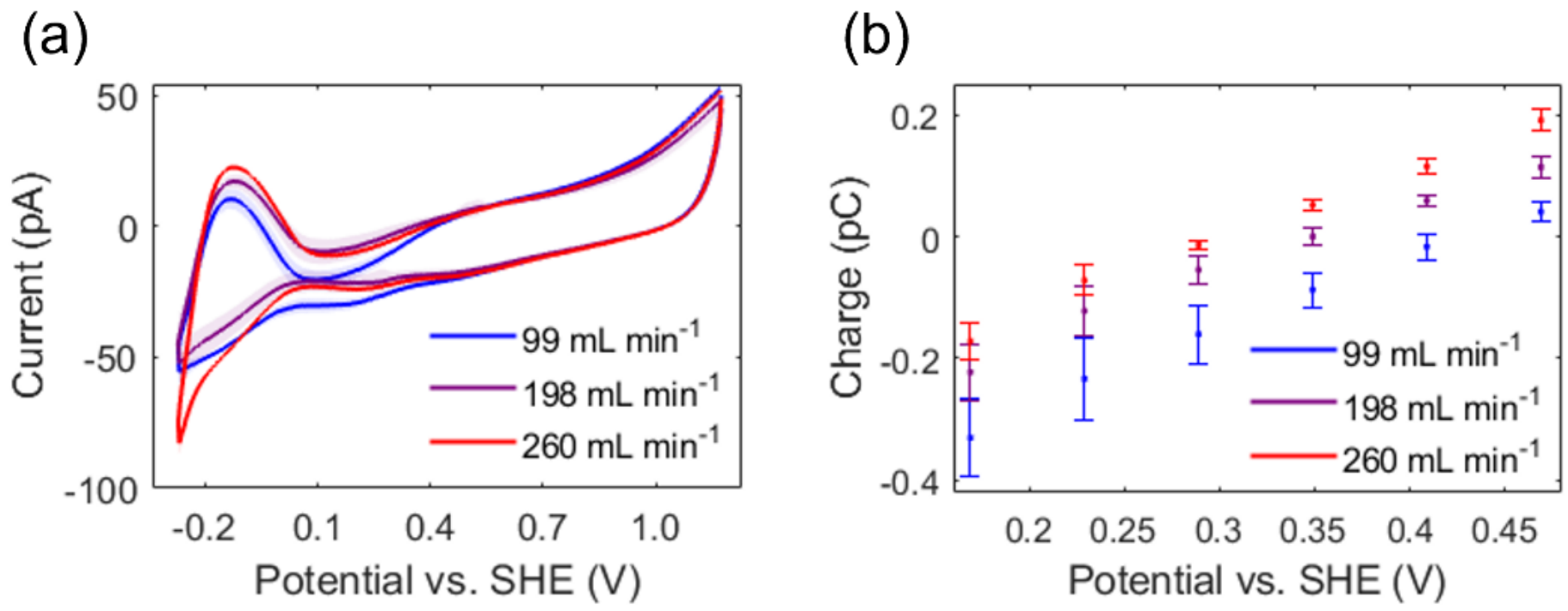

Figure S8. (a) Averaged cyclic voltammograms for Pt under different Ar flow (blue 99 mL min^{-1}, purple 198 mL min^{-1}, red 260 mL min^{-1}). The average was calculated taking the points lying in the regions where each flow was used as marked in Figure 2 of main manuscript, the standard deviation is shown as colored shades. (b) Averaged charge as a function of the electrode potential for the measurements performed under different Ar flow. Standard deviations are represented by error bars.

S5. Supporting Information 4 – Experiments using different conditioning potentials

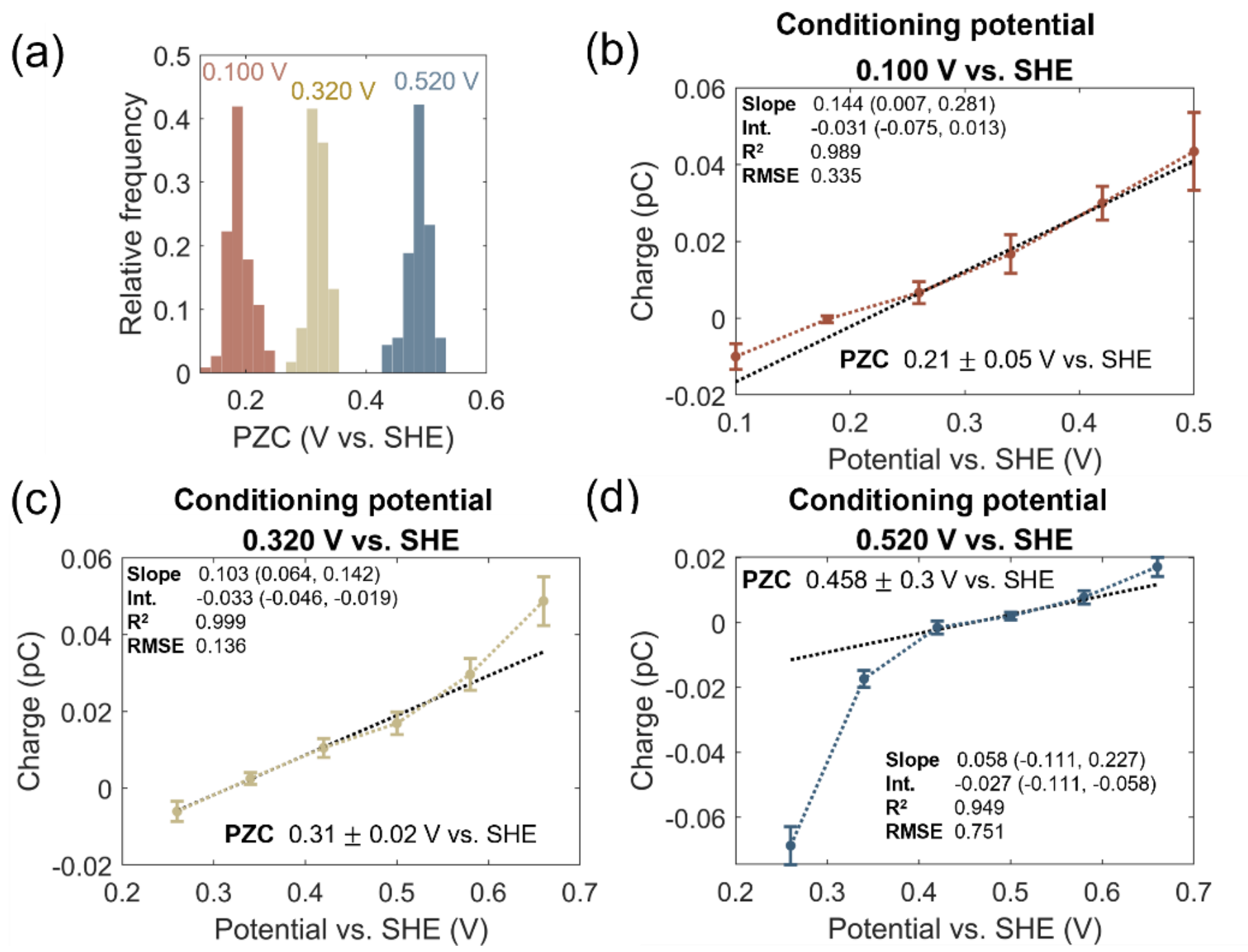

Figure S9. (a) Histogram showing the distribution of the PZC grouped by the conditioning potential: 0.10 V in dark red, 0.32 in yellow and 0.52 V in dark blue (vs. SHE). The numbers of measurements considered were 112, 113 and 90 respectively. Averaged charge as a function of the electrode potential for the measurements performed using different conditioning potentials. (b) 0.100 (c) 0.320 and (d) 0.520 V vs. SHE. Error bars indicate standard deviations. Black dotted lines indicate linear regressions including points within the capacitive region of Pt, while the colored dotted lines serve as visual references to better compare the experimental charge trends with the corresponding linear fits.

S6. Supporting Information 5 – Experiments with different pipette sizes

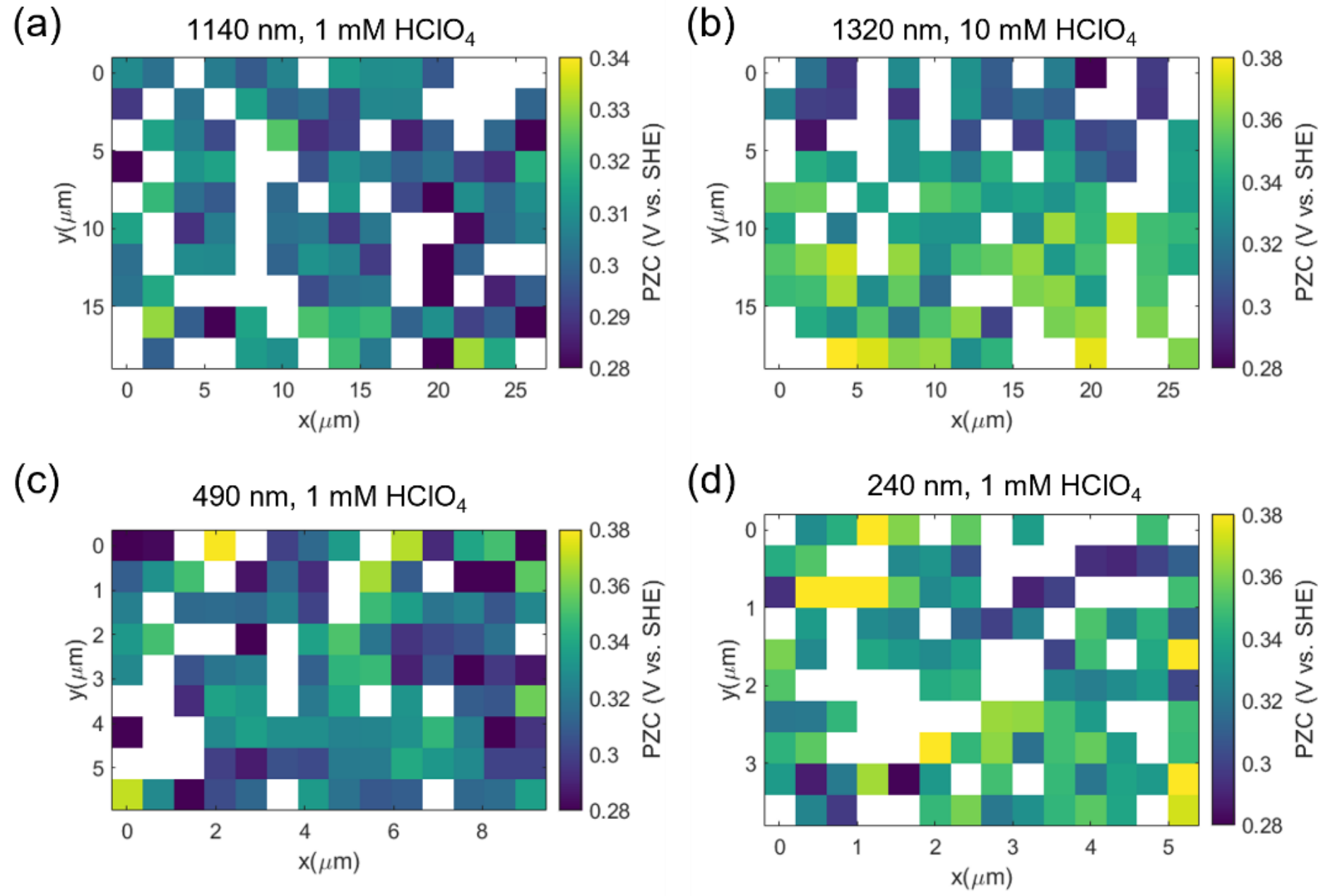

Figure S10. Heatmaps showing the local PZC values of Pt measured using SECCM with pipettes of different diameters: (a) 1140 nm filled with 1 mM $HClO_4$, (b) 1320 nm filled with 10 mM $HClO_4$, (c) 490 nm filled with 1 mM $HClO_4$, and (d) 240 nm filled with 1 mM $HClO_4$. Transparent pixels indicate discarded data points for which the coefficient of determination (R^2) of the PZC fit was less than 0.95.

Table S2. Statistical descriptors of the PZC values obtained from different SECCM measurements, including median, median absolute deviation (MAD), mean, standard deviation (SD) and sample size. PZC values are given in V vs. SHE.

Measurement	Median PZC	MAD PZC	Mean PZC	SD PZC	Sample size
1 mM $HClO_4$, 1140 nm	0.2988 V	0.0078 V	0.299 V	0.0113 V	96
10 mM $HClO_4$, 1320 nm	0.3403 V	0.0116 V	0.341 V	0.0153 V	97
1 mM $HClO_4$, 490 nm	0.3181 V	0.0161 V	0.317 V	0.0247 V	100
1 mM $HClO_4$, 240 nm	03372 V	0.0134 V	0.337 V	0.0254 V	94

Statistical comparison of PZC values obtained in 1 mM and 10 mM $HClO_4$

The difference in median PZC between measurements performed in 1 mM and 10 mM $HClO_4$ is approximately of 0.041 V (0.299 vs. 0.337 V). This shift exceeds the spread of the individual datasets. For the 1 mM measurements, the MAD and SD are 0.0078 V and 0.0113 V, respectively, while for the 10 mM measurements they were 0.116 V and 0.0153 V. The observed shift is therefore approximately 2.7 times larger than the SD of the individual datasets, supporting that the electrolyte induced change in PZC is substantially greater thant the measured variability.

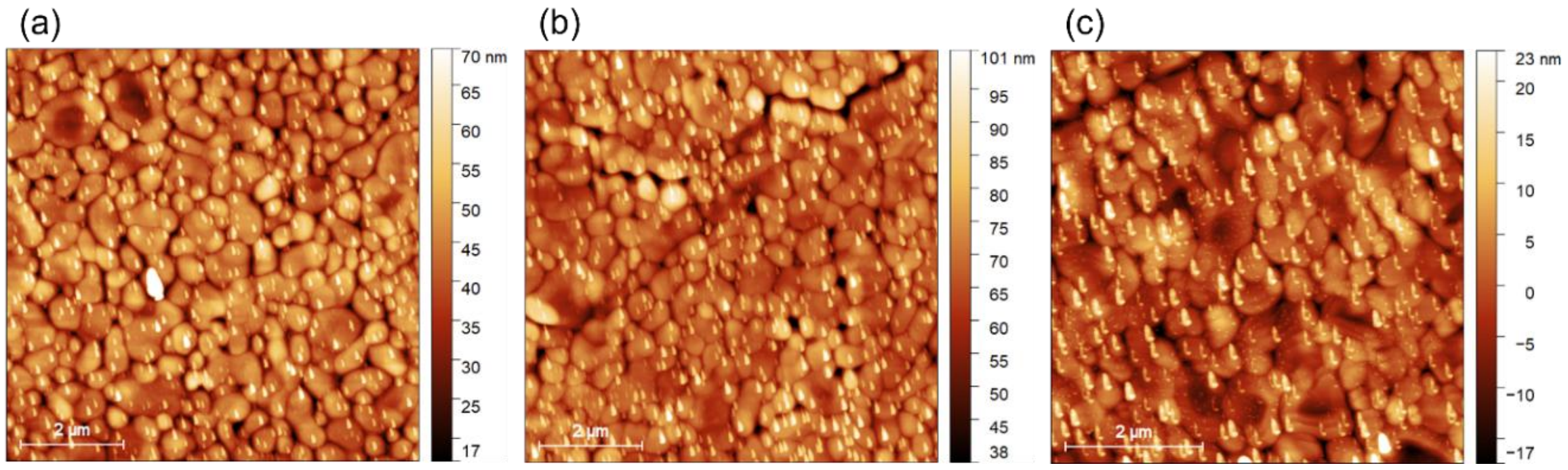

Figure S11. Atomic force microscopy topography of regions close to the SECCM measurement areas. The images reveal a polycrystalline surface morphology with grain lateral dimensions on the order of 100–900 nm. These features are comparable to the characteristic length scales probed by the submicron SECCM nanopipettes (490, and 240 nm). No direct spatial registration between AFM and SECCM measurement points is implied.

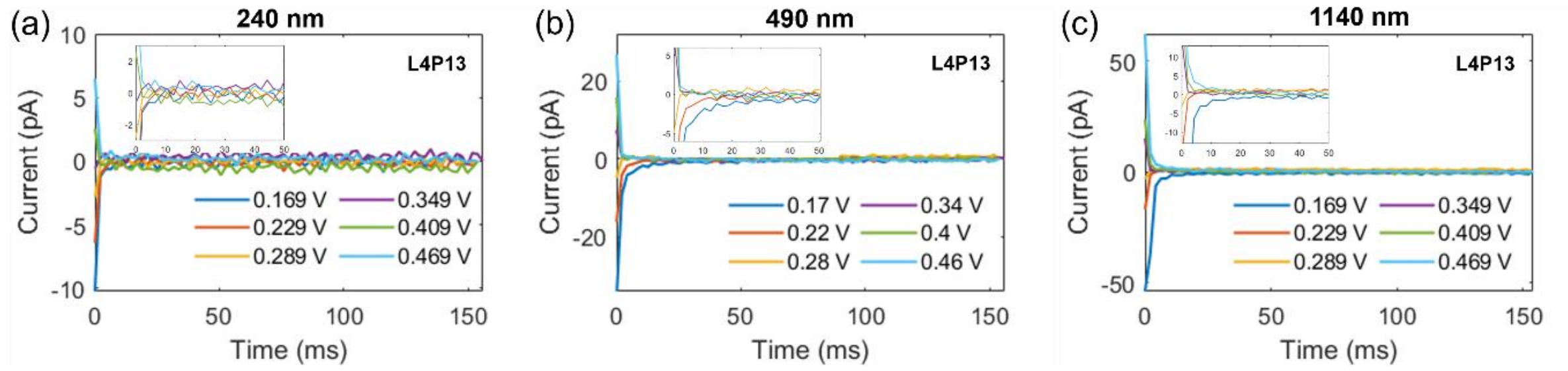

Figure S12. Exemplary current transients recorded during PZC measurements using pipettes with opening tip diameter of (a) 240 nm, (b) 490 nm and (c) 1140 nm.

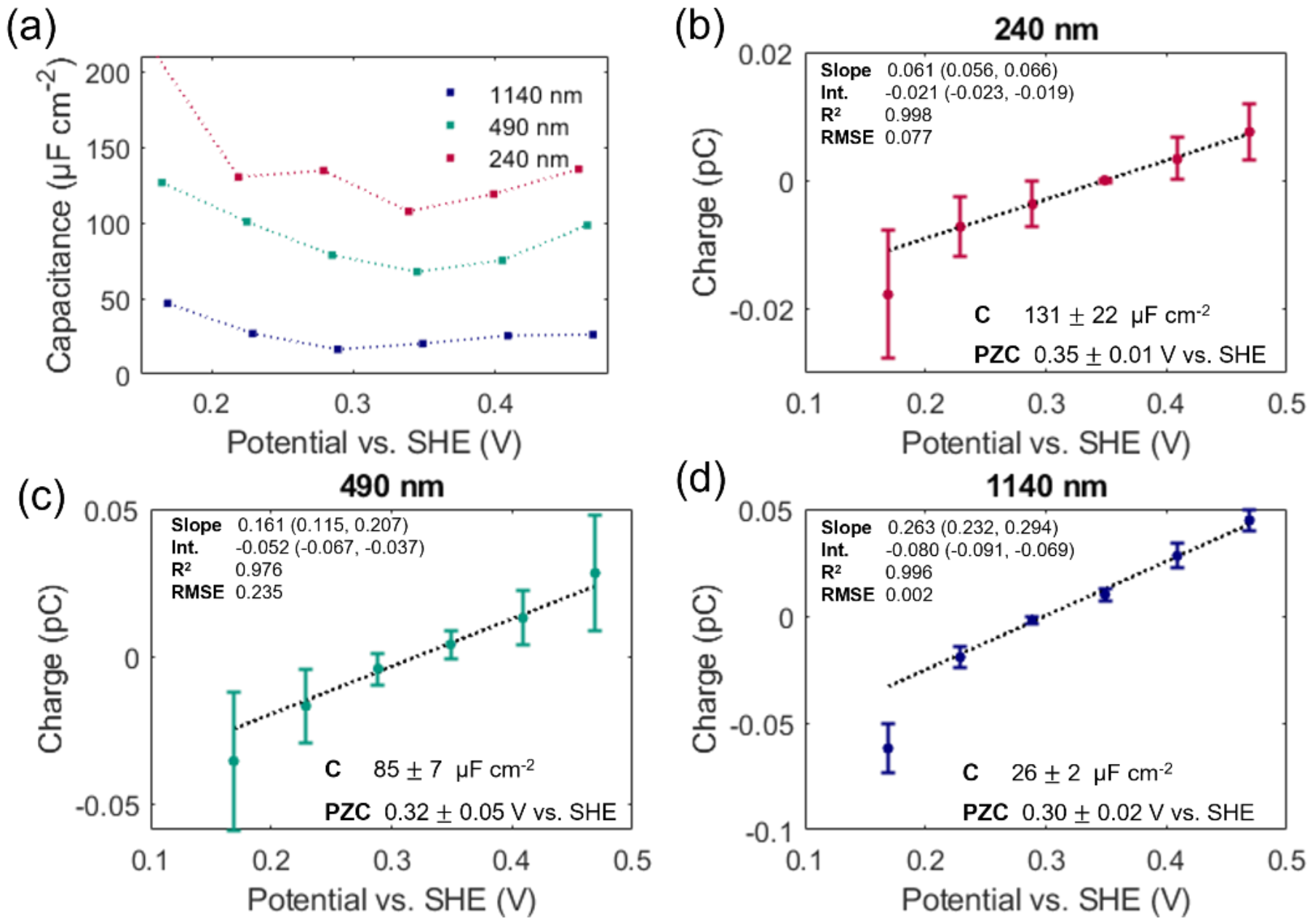

Figure S13. (a) Capacitance calculated with the estimated charges and the SECCM tip opening area as described in section Supporting Information 3. Averaged charge as a function of the electrode potential for measurements performed with pipettes of diameter (b) 240 nm, (c) 490 nm, and (d) 1140 nm. Error bars represent standard deviations. Dotted lines correspond to linear regressions excluding potential points within the Pt hydrogen adsorption region (below 0.2 V vs. SHE). Insets report the regression parameters (slope, intercept, and R^2).

Table S3. Diameter of the wetted area estimated from the H desorption charge and specific capacitance calculated from the slope of the charge vs. potential plots (Figure S10, S8c and 3b) and the estimated wetted area or SECCM tip area. The diameter value reported correspond to the median of the value calculated in each valid measurement.

**Measurement**	**Wetted diameter (nm)**	**specific capacitance (μF cm^{-2})** by wetted area	by tip area
1 mM $HClO_4$, 1140 nm	990	33	25
10 mM $HClO_4$, 1320 nm	1300	27	26
1 mM $HClO_4$, 490 nm	580	61	85
1 mM $HClO_4$, 240 nm	280	96	131
1 mM $HClO_4$, 360 nm	350	107	101

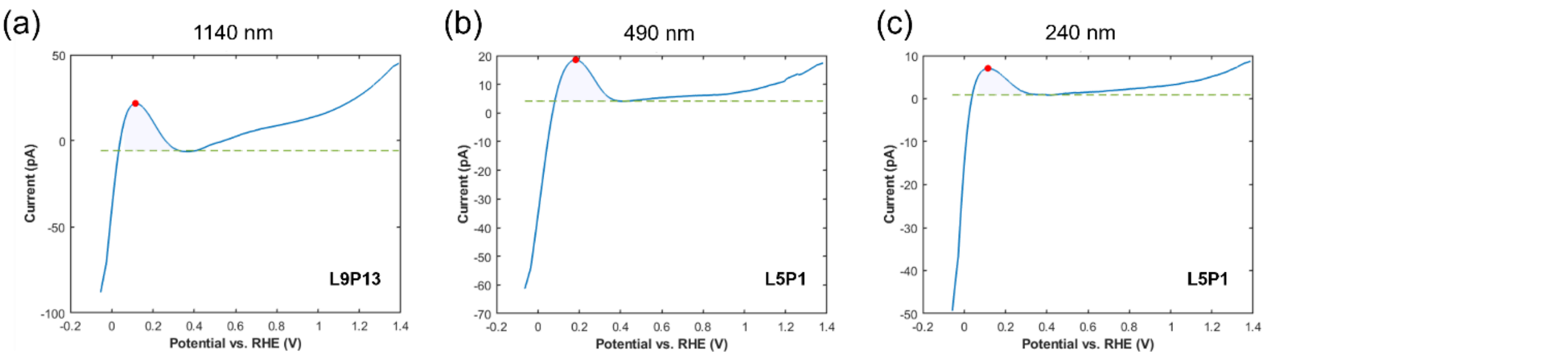

Figure S14. Representative anodic sweeps of the voltammograms recorded on a Pt thin film in contact with 1 mM $HClO_4$ using SECCM nanopipettes with diameters of (a) 1140 nm (b) 490 nm and (c) 240 nm. The traces were used to estimate the wetted area for each SECCM measurement. This was achieved by integrating the charge associated with hydrogen desorption, defined as the area between the anodic sweep and the capacitive baseline over the potential window 0.05 - 0.35 V vs. RHE. The resulting charge was converted into electrochemically active surface area using a specific H adsorption charge of 210 μC/cm^2 for Pt. The corresponding wetted diameter reported in Table S2 was estimated assuming a circular contact geometry.

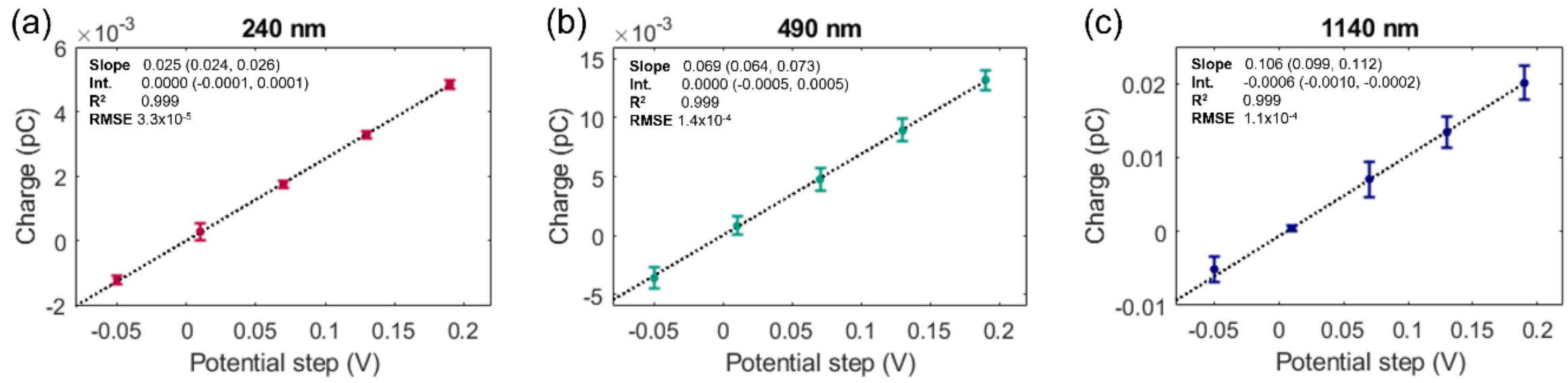

Figure S15. Averaged charge estimated from current transients recorded after a potential step while the pipette was held on the air as a function of the potential step for the measurements performed in (a) 240, (b) 490 nm and (c) 1140 nm. Error bars indicate standard deviations. Dotted lines show the linear fitting and the regression parameters are provided in the insets.

S7. Supporting Information 6 – HER analysis with model fitting

Fitting linear sweep voltammetry data

The cathodic sweep recorded after the PZC measurement was fitted using a model describing the SECCM current as a function of applied potential. Fixed parameter values used in the model are listed in Table S4.

Table S4. Fixed physical and electrochemical parameters used for modeling the cathodic SECCM response following the PZC measurement. Parameters include fundamental constants, electrolyte properties, and experimental geometry.

Faraday constant, F	96485 **C mol^{-1}**
Ideal gas constant, R	8.314 **J mol^{-1} K^{-1}**
Temperature, T	298 **K**
$HClO_4$ bulk concentration, C_b	1×10^{-6} **mol cm^{-3}**
H^+ diffusion coefficient, D_{H^+}	9.3×10^{-5} **cm^2 s^{-1}**
Conductivity 1 mM $HClO_4$, κ	0.00041 **S cm^{-1}**
Electrons transferred, n	1
Pipette radius, r_P	1.8×10^{-5} **cm**

The fitted kinetic parameters are reported as intervals in Table S5. These intervals correspond to 95% confidence intervals obtained using a bootstrap resampling approach. The experimental current–potential data were resampled with replacement and refitted to extract the three model parameters $nFAC_bk_0$, the transfer coefficient α, and the limiting current $i_{\mathrm{lim}}^{\mathrm{SECCM}}$. A total of 500 bootstrap iterations were performed, which was sufficient to yield stable confidence intervals for all fitted parameters. In the table are reported two set of parameters, first ones without considering ohmic drop and the second one considering ohmic drop.

Table S5. Kinetic parameters obtained from voltametric model fitting at different applied potentials versus SHE. Values are reported as 95% bootstrap confidence intervals for the exchange current ($i_0 = nFAC_bk_0$), transfer coefficient (α), SECCM limiting current ($i_{\mathrm{lim}}^{\mathrm{SECCM}}$), and standard rate constant (k_0). The coefficient of determination (R^2) reflects the goodness of fit.

Without ohmic drop			
	**At 0.10 V vs- SHE**	**At 0.32 V vs. SHE**	**At 0.52 V vs. SHE**
$i_{0=}nFAC_bk_0$ (pA)	[1.95, 1.97]	[2.32, 2.34]	[2.05, 2.07]
Transfer coefficient, α	[0.843, 0.845]	[0.711, 0.713]	[0.732, 0.734]
Limiting current, i_{lim}^{SECCM} (pA)	[-150.1, -150.2]	[-191.4 -191.5]	[-173.5 -173.6]
Coefficient of determination, R^2	0.9997	0.9998	0.9998
Considering ohmic drop			
$i_{0=}nFAC_bk_0$ (pA)	[1.21, 1.22]	[1.52, 1.53]	[1.41, 1.42]
Transfer coefficient, α	[1.029, 1.031]	[0.847, 0.849]	[0.860, 0.862]
Limiting current, i_{lim}^{SECCM} (pA)	[-150.1 -150.2]	[-191.4 -191.5]	[-173.5 -173.6]
Standard rate constant, k_0 (cm s^{-1})	[0.0123, 0.0124]	[0.0155, 0.0156]	[0.0143, 0.0144]
Coefficient of determination, R^2	0.9995	0.9998	0.9996
